# Adaptation strategies and collective dynamics of extraction in networked commons of bistable resources

**DOI:** 10.1038/s41598-021-01314-2

**Published:** 2021-11-09

**Authors:** Andrew Schauf, Poong Oh

**Affiliations:** Wee Kim Wee School of Communication and Information, Nanyang Technological University, Singapore, 639798 Singapore

**Keywords:** Ecological networks, Complex networks, Social evolution, Psychology and behaviour, Socioeconomic scenarios

## Abstract

When populations share common-pool resources (CPRs), individuals decide how much effort to invest towards resource extraction and how to allocate this effort among available resources. We investigate these dual aspects of individual choice in networked games where resources undergo regime shifts between discrete quality states (viable or depleted) depending on collective extraction levels. We study the patterns of extraction that emerge on various network types when agents are free to vary extraction from each CPR separately to maximize their short-term payoffs. Using these results as a basis for comparison, we then investigate how results are altered if agents fix one aspect of adaptation (magnitude or allocation) while letting the other vary. We consider two constrained adaptation strategies: uniform adaptation, whereby agents adjust their extraction levels from all CPRs by the same amount, and reallocation, whereby agents selectively shift effort from lower- to higher-quality resources. A preference for uniform adaptation increases collective wealth on degree-heterogeneous agent-resource networks. Further, low-degree agents retain preferences for these constrained strategies under reinforcement learning. Empirical studies have indicated that some CPR appropriators ignore—while others emphasize—allocation aspects of adaptation; our results demonstrate that structural patterns of resource access can determine which behavior is more advantageous.

## Introduction

*Common-pool resources* (CPRs) are rivalrous: that is, one individual’s use of the resource diminishes the benefits available to others, leading to a conflict between individual and collective interests. Communities that share CPRs, however, are often able to collectively resolve these dilemmas by coordinating their individual actions in ways that sustain resource quality more effectively than if they were regulated by centralized authorities^1,2^. To better understand the mechanisms behind this self-regulation, previous studies have examined how individuals from different socio-ecological contexts adapt to changing resource conditions^3–8^. Some studies engaged real-life CPR users from diverse backgrounds in experiments designed to model the dilemmas encountered when sharing CPRs such as fisheries or pastures^4–6^. In these games, excessive extraction from a CPR causes its depletion; by reducing extraction, the resource’s quality can then be restored. Players were informed of the quality of multiple resources in each round, and then prompted to choose how much to extract from each in the next round. Ostensibly, players had no short-term incentive to *reduce* their extraction in a way that would allow for a resource’s renewal. Instead, rational individuals were expected to *reallocate* extraction away from a depleted resource and onto the other, collectively exhibiting oscillatory “rotation”^5^ between resources over time, as predicted elsewhere by computational models^9^. However, contrary to expectation, real-life CPR users tended to react to the depletion of *one* resource by reducing extraction *everywhere*, forgoing potential payoffs by alleviating pressure on all resources^5^. If CPR users tend to view the depletion of one resource as a warning signal to reduce their overall extraction in this way, then this potentially serves to prevent a more widespread cascade of resource depletion from following. So, in this context, behavior that reconciles an individual’s self-interest with the longer-term sustainability of the commons might come in the form of this kind of innate adaptive tendency, rather than through obedience to some predefined “cooperative” rules that restrict an individual’s extraction. The specific ways that individuals adapt to changing resource conditions within a structured commons—whether they tend to reallocate, or rather to adjust their extraction levels more uniformly across multiple resources—could thus play a crucial role in determining a population’s capacity for collective self-regulation.

While framing the adaptive behavior of CPR appropriators in terms of these dual aspects (allocation versus overall magnitude of effort), these previous studies presented empirical observations suggesting that an individual’s tendency to either emphasize or ignore the allocation aspects of extraction depends on the unique social and ecological conditions within which the individual resides. This raises evolutionary questions: *What causes individuals to develop behavior that deviates from “individual payoff-maximizing behavior”*^5^? We hypothesize that the *structural patterns of resource access* within a population represent one aspect of the socio-ecological environment that shapes these individual tendencies. Discourse about the commons sometimes alludes to the idea that self-regulation is related to a resource’s internal structure and heterogeneity, for example describing the commons as “local systems that are spatially diverse, even in the same period of time, and precisely for this reason, … their diversity and flexibility enable the best use of natural resources upon which the commons depend, avoiding over-exploitation, degradation and destruction.”^10^ Recent experimental work has investigated the role of spatial structure in cooperation by groups of CPR appropriators^11^, while other empirical studies have applied network analysis to study patterns of information exchange between CPR-sharing communities^12^. Recent computational studies have investigated the role of social network structure for agents sharing a single CPR^13,14^. When the connections between individual agents and multiple CPRs are described by affiliation networks, the efficiency of extraction by rational agents has been shown to be determined by network degree heterogeneity^15,16^. If these agents view resource extraction purely as an allocation problem, attempting to increase their individual payoffs by shifting extraction between resources without changing the overall amount of effort exerted, then they achieve more optimal outcomes at the population level^16^. On the other hand, it seems that if agents lacked the flexibility to vary their extraction in terms of allocation, they could suffer large losses in collective wealth.

The connection between these abstract network-based models and real-world CPRs remains tenuous, though, since their conclusions hinge upon strong assumptions about individual behavior. In a networked CPR model mentioned above, for instance, agents are made to obey a *fixed-magnitude* assumption, varying their extraction levels only in terms of allocation. This kind of limitation is by no means unique to this networked CPR model, as it parallels a *fixed-allocation* assumption typically adopted in networked public goods games (PGGs) including the multiplayer Prisoner’s Dilemma^17,18^: agents must identify as either “cooperators” or “defectors” consistently across *all* goods. Although several studies have allowed for some amount of adaptative allocation of contributions by cooperators^19–24^, if this cooperator/defector dichotomy is discarded altogether, then voluntary contribution behavior no longer spreads via imitation through a social network (Supplementary Information S6). These networked evolutionary game models all share a common feature: one or the other aspect of agents’ choice—either magnitude or allocation—is effectively fixed. Meanwhile, these models have not always been successful in explaining the collective behavior of real-life human players^25^. A deeper understanding of these assumptions is necessary to link these network models to real-world dilemmas in more meaningful ways. The present work aims to address this gap.

**Figure 1:**
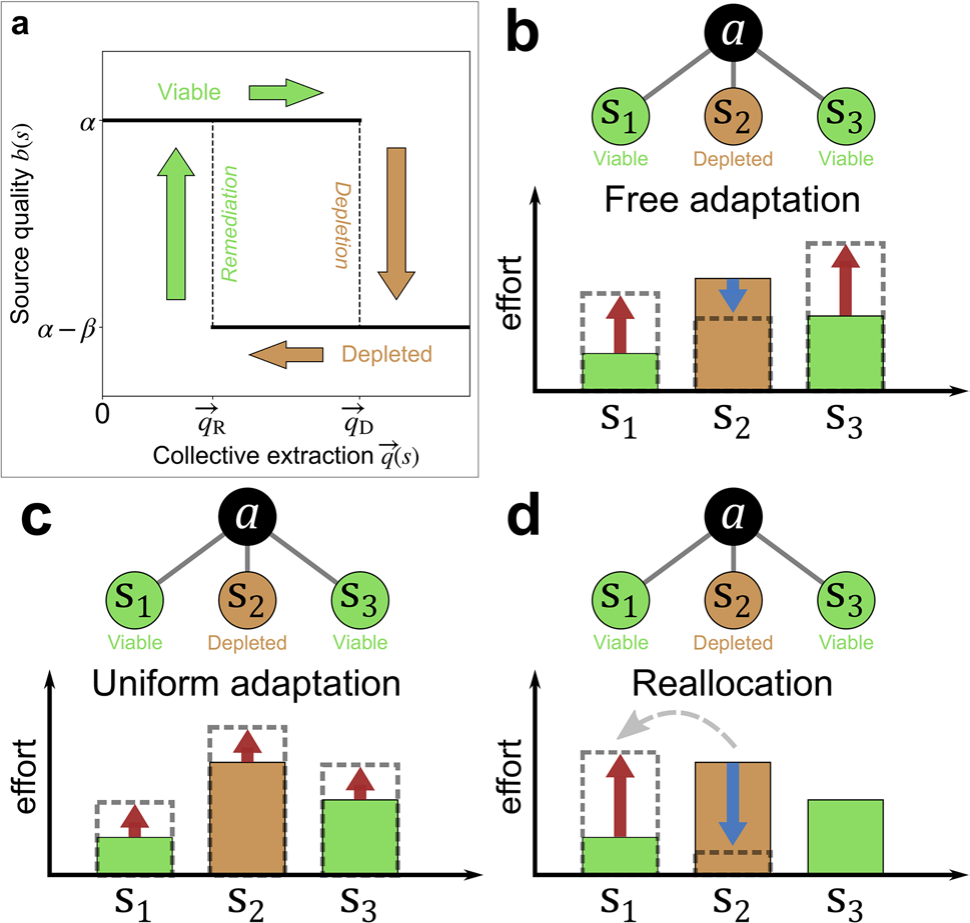
*Evolution of resource quality and agent extraction:* (**a**) Source quality exhibits bistability and hysteresis with respect to collective extraction; *Illustration of three update rules.* An agent a, linked to two viable sources (${s}_{1}$ and ${s}_{3}$) and one depleted source (${s}_{2}$) adjusts its extraction levels: (**b**) Under *free adaptation*, the agent increases its extraction from viable sources and decreases its extraction from depleted sources, each at rates proportional to the expected payoff increase associated with the adjustment. (**c**) Under *uniform adaptation*, the agent is constrained to increase (or decrease) its extraction from all sources by the same amount, at a rate that depends on the mean quality of the three sources. (**d**) Under *reallocation*, the agent shifts an increment of effort from a depleted source to a randomly selected viable source; the agent’s total individual extraction is conserved.

In doing so, we adopt a *bistable* model of CPR quality: a resource immediately becomes depleted when total extraction exceeds a *depletion threshold*, but is restored to its original state after total extraction drops below a *remediation threshold* (Fig. 1a). This generalizes the model used in the aforementioned experimental games^4–6^ onto systems with greater numbers of resources and agents, whose interconnectedness is described by complex networks. Besides having these precedents in experiments, this model recalls a variety of ecological systems that have indeed been found to undergo these kinds of sudden regime shifts, analogous to the phase transitions studied in statistical mechanics, due to positive feedback mechanisms that lead to bistability^26–29^. Examples include common-pool resources such as fisheries^30,31^, water resources^32–34^, and pastures or forests^35^. By modelling these tipping-point phenomena, the current model can be seen as a CPR parallel to public goods games such as the multiplayer Stag Hunt^36^. In these games, the benefits provided by a public good undergo sudden, discontinuous transitions to higher values, rather than varying smoothly with respect to players’ collective contributions. Without purporting that all real-world CPR dilemmas can be covered by a few general networked models^37^, the current study complements previous work on networked games of linearly degrading CPRs^15,16^ by addressing a wide range of phenomena that those models neglect: resources that exhibit highly nonlinear, asymmetric depletion and recovery behavior.

With the above motivations in mind, we introduce a model of resource extraction dynamics on affiliation networks that link a population of agents to a set of bistable common-pool resources. At each round of an iterated game, agents survey current resource conditions and then adjust their extraction levels accordingly at rates proportional to the immediate increases in individual payoffs they expect to achieve thereby. Agents channel this short-sighted self-interest through one of three incremental update rules: (1) *free adaptation*, whereby agents vary their extraction levels from each CPR separately with no constraint; (2) *uniform adaptation*, whereby agents are constrained to adjust their extraction levels at all CPRs by the same amount; or (3) *reallocation*, whereby agents shift their extraction away from depleted CPRs under the constraint that their total extraction remains constant (Fig. 1b–d). ***How do the structural patterns of resource access within a networked commons shape the extraction patterns and wealth distributions that emerge?*** To address this question, we first investigate the dynamics of free adaptation on various bipartite networks having different levels of degree heterogeneity among both agents and CPR nodes. In the constantly oscillating extraction dynamics that result from CPR bistability, we find that greater degree heterogeneity leads to reduced average extraction, reduced resource quality, and reduced collective wealth under free adaptation. This simultaneous reduction in both extraction *and* resource quality, which appears to contradict the rivalrous property of common-pool resources, demonstrates that sudden-onset changes in resource conditions can lead to drastically different emergent outcomes than if conditions change more gradually.

Having described the patterns of extraction that emerge in the absence of constraints imposed upon agents’ adaptation behavior, we then study how these extraction patterns are altered when constraints are introduced. ***Will a widespread adoption of constrained adaptation behavior offer some advantage to a population?*** We simulate dynamics in which all members of a population practice the same mixed strategy that combines free adaptation with the constrained update rules: *uniform adaptation* and *reallocation*. In networks with uniform degree, populations practicing free adaptation achieve greater collective wealth than those that sometimes apply constrained update rules. However, in networks with greater degree heterogeneity, strategies that incorporate uniform adaptation can achieve greater collective wealth. Having studied how agents’ innate adaptation strategies can affect extraction dynamics, we then allow dynamics, in turn, to influence agents’ adaptation strategies. ***Will individuals develop preferences for constrained adaptation behavior over unconstrained adaptation?*** Using generalized reinforcement learning^38^ to simulate how individuals might change their adaptation behavior based on experience, we observe that higher-degree agents tend to quickly “unlearn” any preferences for uniform adaptation or reallocation in favor of the greater flexibility of free adaptation. However, lower-degree agents, for whom the distinction between these different adaptation strategies is less pronounced, can retain preferences for these additional constraints. Together, these results provide novel evolutionary demonstrations that the structural patterns of resource access within a community, and individuals’ positions within these structures, can shape how CPR appropriators learn to adapt to changing conditions.

## Methods

### Agent-resource affiliation networks

We consider games involving populations of *agents* that extract from multiple common-pool *sources* (which term we use for nodes representing resources in accord with previous related work^15,16^). Agents’ access to sources is defined by bipartite networks, wherein a link between an agent and a source indicates that the agent can access that source. This access is determined by some exogenous factors and remains fixed in time. The set of agents affiliated with a particular source $s$ is denoted as ${\mathbf{A}}_{s}$, while the set of sources affiliated with a particular agent $a$ is denoted as ${\mathbf{S}}_{a}$. The degree of an agent $a$ is denoted by $m(a)$, and the degree of a source $s$ by $n(s)$.

**Figure 2:**
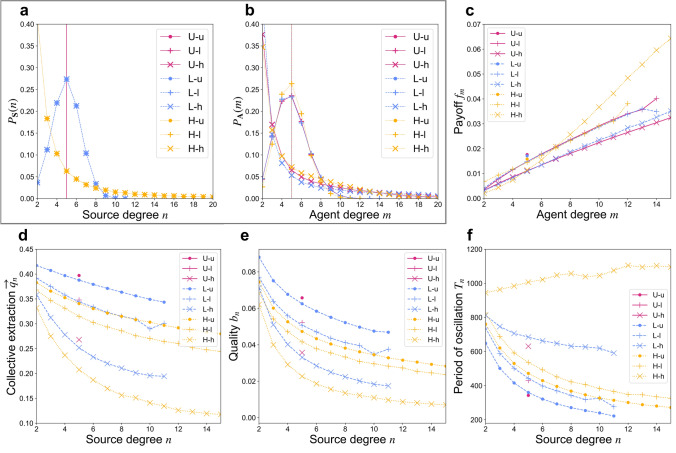
(**a**) *Source degree distributions* and (**b**) *Agent degree distributions* for 9 network ensembles, each representing a combination of a Uniform-degree (U), Low-heterogeneity (L), or High-heterogeneity (H) source degree distribution with a uniform-degree (u), low-heterogeneity (l), or high-heterogeneity (h) agent degree distribution. Ensemble mean time-averaged quantities from pure free adaptation dynamics: (**c**) *Agent payoffs* $f(a)$ as a function of agent degree $m(a)$; (**d**) *Collective extraction* $\overrightarrow{q}(s)$ as a function of source degree $n(s)$; (**e**) *Source quality* $b(s)$ as a function of source degree ﻿﻿$n(s)$; and (**f**) *Period of oscillation* $T(s)$ as a function of source degree $n(s)$. Means are computed from simulations on ${10}^{3}$ networks of each type.

To explore the effects of network topology upon extraction dynamics and wealth distributions, we generate ensembles of ${10}^{3}$ networks, each having $50$ agents and $50$ sources and sharing mean agent degree $\langle m\rangle =5$ and mean source degree $\langle n\rangle =5$. All networks thus share the same total numbers of agents, sources, and links, but differ in how these links are distributed among agents and sources. We generate 9 network ensembles, each generated to represent a particular combination of one of three types of degree heterogeneity in its source degree distribution (**U**: uniform-degree, **L**: low-heterogeneity, or **H**: high-heterogeneity) with one of three similar distributions of agent degree (**u**, **l**, or **h**^39^) (Supplementary Information S1.1). Degree histograms, averaged over each ensemble, provide a representative *source degree distribution* ${P}_{\mathbf{S}}(n)$ and *agent degree distribution* ${P}_{\mathbf{A}}(m)$ for each network type (Fig. 2a and b). It is worth noting that the results of the simulations depend primarily on the degree distributions of agents and sources rather than on the overall size of the networks used (Supplementary Information S3.1).

### Networked CPR extraction game

On these networks, we simulate iterative games in which agents vary the extraction effort that they apply to their affiliated sources, altering the quality of these sources; in turn, these changes in source quality then influence how agents adapt their extraction levels in subsequent rounds. The ***extraction effort*** exerted by agent $a$ upon its affiliated source $s$ is denoted as $q(a,s)$. The total effort exerted by an agent $a$, its ***individual extraction,*** is denoted by $\overleftarrow{q}\left(a\right)=\sum_{s\in {\mathbf{S}}_{a}}q(a,s)$. The total effort exerted upon source $s$, or its ***collective extraction***, is denoted by $\overrightarrow{q}\left(s\right)=\sum_{a\in {\mathbf{A}}_{s}}q(a,s)$. The ***quality*** of a source $s$ is quantified by the benefit $b(s)$ per unit extraction effort applied that the source provides. The cost associated with extraction is given by a convex (quadratic) function of $\overleftarrow{q}\left(a\right)$, such that marginal costs increase with individual extraction^15,16^. In addition to modelling the increasing costs (i.e., diminishing returns) associated with the physical act of extraction itself, this could also reflect escalating, informal social penalties that result from increasing extraction (i.e., “graduated sanctions”^1,40^). The net ***payoff*** accumulated by an agent $a$ in a game iteration is thus

1 $$f\left( a \right) = \left[ {\mathop \sum \limits_{{s \in {\mathbf{S}}_{a} }} q\left( {a,s} \right) \cdot b\left( s \right)} \right] - \frac{\gamma }{2}{ }\mathop{q}\limits^{\leftarrow} \left( a \right)^{2} ,$$

where $\gamma$ is a positive cost parameter.

### Bistable model of CPR depletion and remediation

Sources are *bistable*, meaning that at any time they can occupy one of two states: (1) a ***viable state***, during which the source provides a benefit of magnitude $\alpha$ in return for each unit of extraction effort, and (2) a ***depleted state***, during which this benefit is reduced by $\beta$ ($0<\beta \le \alpha$). Sources immediately transition from viable to depleted states when the collective extraction surpasses a ***depletion threshold*** ${\overrightarrow{q}}_{\mathrm{D}}(s)$. Depleted sources may then transition to active states again when collective extraction falls below a ***remediation threshold*** ${\overrightarrow{q}}_{\mathrm{R}}(s)$ (Fig. 1a). Source quality is thus given by

2 $$b\left( s \right) = \alpha - \beta \chi \left( s \right),$$

where $\chi \left( s \right) = 0$ for viable sources and $\chi \left( s \right) = 1$ for depleted sources. The state $\chi \left( s \right)$ of a source thus evolves such that at an iteration $t$ (we index iterations by subscripts where relevant) by

3 $$\chi_{t} \left( s \right) = \left\{ {\begin{array}{lll} {1, } & {{\text{if }} \chi_{t - 1} \left( s \right) = 0{\text{ and }}\vec{q}_{t} \left( s \right) > \vec{q}_{{\text{D}}} \left( s \right)} \\ {0, } & {{\text{if }} \chi_{t - 1} \left( s \right) = 1{\text{ and }}\vec{q}_{t} \left( s \right) \le \vec{q}_{R} \left( s \right)} \\ {\chi_{t - 1} \left( s \right),} & {\text{otherwise }} \\ \end{array} } \right.$$

In the results that follow, we focus upon a *uniform capacity* scenario, wherein all sources share identical threshold values $\vec{q}_{{\text{D}}} \left( s \right) \equiv \vec{q}_{{\text{D}}}$ and $\vec{q}_{{\text{R}}} \left( s \right) \equiv \vec{q}_{{\text{R}}} \left( s \right)$. An alternative *degree-proportional capacity* scenario, in which threshold values increase with source degree, is discussed in the Supplementary Information (S3.4.2).

### Free adaptation

Under the free adaptation strategy, an agent updates its extraction levels independently at each of its affiliated sources depending on the state of each (Fig. 1b). As in the replicator rule often applied in networked evolutionary game models^17,41,42^, the rate at which an agent adapts its extraction levels within a time interval ${\Delta }t$ is proportional to the marginal payoff that the agent expects to attain thereby:

4 $$\frac{{{\Delta }q\left( {a,s} \right)}}{{{\Delta }t}} = k\frac{\partial f\left( a \right)}{{\partial q\left( {a,s} \right)}},$$

where $k$ is a rate constant. So, each extraction level $q\left( {a,s} \right)$ is updated according to

5 $$q_{t + 1} \left( {a,s} \right) = q_{t} \left( {a,s} \right) + k\left[ {\alpha - \beta \chi_{t} \left( s \right) - \gamma {\mathop{q}\limits^{\leftarrow}}_{t} \left( a \right)} \right].$$

The higher an agent’s individual extraction $\overleftarrow{q}(a)$, the more slowly it will increase its extraction from viable sources, and the more rapidly it will reduce its extraction from depleted sources.

### Uniform adaptation

When applying the uniform adaptation strategy, an agent adjusts each of its extraction levels by the same magnitude $\Delta q\left(a,s\right)\equiv\Delta \overleftarrow{q}(a)/m\left(a\right)$ (Fig. 1c). Assuming again that the rate at which an agent enacts this update is proportional to the associated marginal payoff, an agent adapts its extraction levels at all of its affiliated sources $s$ by

6 $$q_{t + 1} \left( {a,s} \right) = q_{t} \left( {a,s} \right) + k\left[ {\alpha - \beta \overline{\chi }\left( a \right) - \gamma {\mathop{q}\limits^{\leftarrow}}_{t} \left( a \right)} \right],$$

where $\overline{\chi }\left( a \right) = \left[ {\mathop \sum \nolimits_{{s^{\prime} \in {\mathbf{S}}_{a} }} \chi \left( {s^{\prime}} \right)} \right]/m\left( a \right)$ is the mean state of the agent’s affiliated sources.

### Reallocation

When practicing reallocation, an agent shifts an increment of extraction effort from a depleted source to a viable source such that its overall individual extraction $\mathop{q}\limits^{\leftarrow} \left( a \right)$ remains unchanged (Fig. 1d). The agent thus randomly selects one depleted source $s_{{\text{D}}} \in {\mathbf{S}}_{a}$ and one viable source $s_{{\text{V}}} \in {\mathbf{S}}_{a}$, if available. Since the marginal payoff per unit reallocated is $\beta$, updates its extraction levels such that

7 $$q_{t + 1} \left( {a,s} \right) = \left\{ {\begin{array}{*{20}c} {q_{t} \left( {a,s} \right) - k\beta , } & {{\text{if}} s = s_{{\text{D}}} } \\ {q_{t} \left( {a,s} \right) + k\beta , } & {{\text{if}} s = s_{{\text{V}}} } \\ {q_{t} \left( {a,s} \right),} & {\text{otherwise }} \\ \end{array} } \right.$$

When an agent’s affiliated sources all share the same quality value, no such reallocation is possible, and so the agent retains its present extraction levels: $q_{t + 1} \left( {a,s} \right) = q_{t} \left( {a,s} \right)$ for all $s \in {\mathbf{S}}_{a}$.

### Mixed strategies

An agent’s ***adaptation strategy*** $$({p}_{0},{p}_{\updownarrow },{p}_{\leftrightarrow })$$ comprises the probabilities that it will practice each of these update rules in any given round: its *free adaptation propensity* ($${p}_{0})$$, its *uniform adaptation propensity* ($${p}_{\updownarrow })$$, and its *reallocation propensity* ($${p}_{\leftrightarrow }$$). An agent’s choice of a particular update rule is thus based only on its own innate inclinations, but the *rate* at which it enacts the selected rule is influenced by current resource conditions. We first simulate dynamics in which the same adaptation strategy is shared by all members of a population throughout the entire course of a simulation. We then consider games in which agents’ individual adaptation strategies are each allowed to independently evolve under generalized reinforcement learning^38,43^ (Supplementary Information S1.3.4). That is, after enacting a chosen update rule in an iteration $$t$$, each agent $$a$$ observes the payoff change $$\Delta {f}_{t}\left(a\right)={f}_{t}\left(a\right)-{f}_{t-1}(a)$$. If $$\Delta {f}_{t}\left(a\right)>0$$, then the agent’s relative propensity to practice this update rule in subsequent rounds is increased. If the agent’s payoffs decreased ($$\Delta {f}_{t}\left(a\right)<0$$), then its propensity to apply the given update rule is reduced accordingly.

### Simulations

In simulations of the CPR extraction game, each iteration involves the following steps:

1. **Agents collect payoffs** based on current extraction levels and resource conditions (Eq. 1).
2. Agents are randomly selected for update, each with probability $u$.
3. Each updating agent **select an update rule** based on its adaptation strategy $({p}_{0} ,{p}_{\updownarrow },{p}_{\leftrightarrow })$.
4. Each updating agent **adjusts its extraction levels** in accord with the chosen update rule (Eqs. 5–7).
5. If applicable, updating agents **adjust their adaptation strategies** using reinforcement learning.
6. **The state **${\varvec{\chi}}({\varvec{s}})$** of each source is updated** based on the new extraction levels (Eq. 3).

Although the update rules﻿ of Eqs. (5–7) deterministically specify the changes an agent will make when enacting a certain update rule under a given set of resource conditions (Step 4), each iteration involves randomness in the set of agents that update (Step 2), in the selections of update rules by each updating agent (Step 3), and in the choices of sources involved in reallocation moves (Eq. 7).

In the simulation results presented below, all sources are set to share $\alpha =\beta =1$, ${\overrightarrow{q}}_{\mathrm{D}}=1$, and ${\overrightarrow{q}}_{\mathrm{R}}=.001$, and initial state $\chi \left(s\right)=0$. All agents share $u=.5$, $k=.02$, and $\gamma =0.2$ unless otherwise noted. The parameter settings considered here ($\beta =\alpha$) represent complete, catastrophic resource depletion. This choice simplifies our analyses by ensuring that agents practicing free adaptation (Eq. 6) will always reduce their extraction from a depleted source and eventually trigger its remediation, regardless of the choice of the cost parameter $\gamma >0$ or remediation threshold ${\overrightarrow{q}}_{\mathrm{R}}\left(s\right)$. That is, all resource depletion events are assumed to be extreme enough to motivate agents to continuously “self-regulate” by remediating depleted sources (see Supplementary Information S5 for a more thorough discussion of these parameter settings).In simulations where reinforcement learning is applied, all agents are initialized with ${p}_{\updownarrow }={p}_{\leftrightarrow }=.333$. For pure free adaptation simulations (${p}_{0}=1$), initial extraction levels were randomized (${q}_{t=0}\left(a,s\right)\in [0,\frac{{\overrightarrow{q}}_{\mathrm{D}}\left(s\right)}{n\left(s\right)}]$). All other simulations (${p}_{0}<1$) were then initialized by setting each agent’s individual extraction to its average value from the free adaptation simulation, ${\overline{\overleftarrow{q }\left(a\right)}}_{0}$, allocated equally among its affiliated sources (${{q}_{t=0}(a,s)=\overline{\overleftarrow{q }\left(a\right)}}_{0}/m(a)$). Simulations were iterated through ${10}^{5}$ steps, and time-averaged quantities were computed over the final $8\times {10}^{4}$ iterations: e.g., $\overline{q }(a,s)=\left(\frac{1}{8\times {10}^{4}}\right){\sum }_{t=2\times {10}^{4}}^{{10}^{5}}{q}_{t}(a,s)$. This duration was chosen based on inspection of simulation results to ensure that free adaptation extraction levels would have settled into steady ranges, and that mixed-strategy and reinforcement learning dynamics (where applicable) would have approached their stable mean values, after an initial transient period.

## Results

### Agent degree heterogeneity is associated with reduced extraction *and* resource quality under free adaptation

When agents practice free adaptation, they increase extraction from viable sources until the sources become depleted and reduce extraction from depleted sources until the sources become remediated. Sources perpetually alternate between viable and depleted states, and no static steady state is reached. Free adaptation represents equilibrium-seeking behavior (Supplementary Information S1.3.1), and thus the time-averaged quantities that characterize these cyclical depletion-remediation dynamics can be considered as a baseline for comparison analogous to Nash equilibrium. We now focus on how each of these quantities depend on an agent or source node’s degree, and how this degree dependence is determined by the degree heterogeneity of the surrounding network.

**Figure 3:**
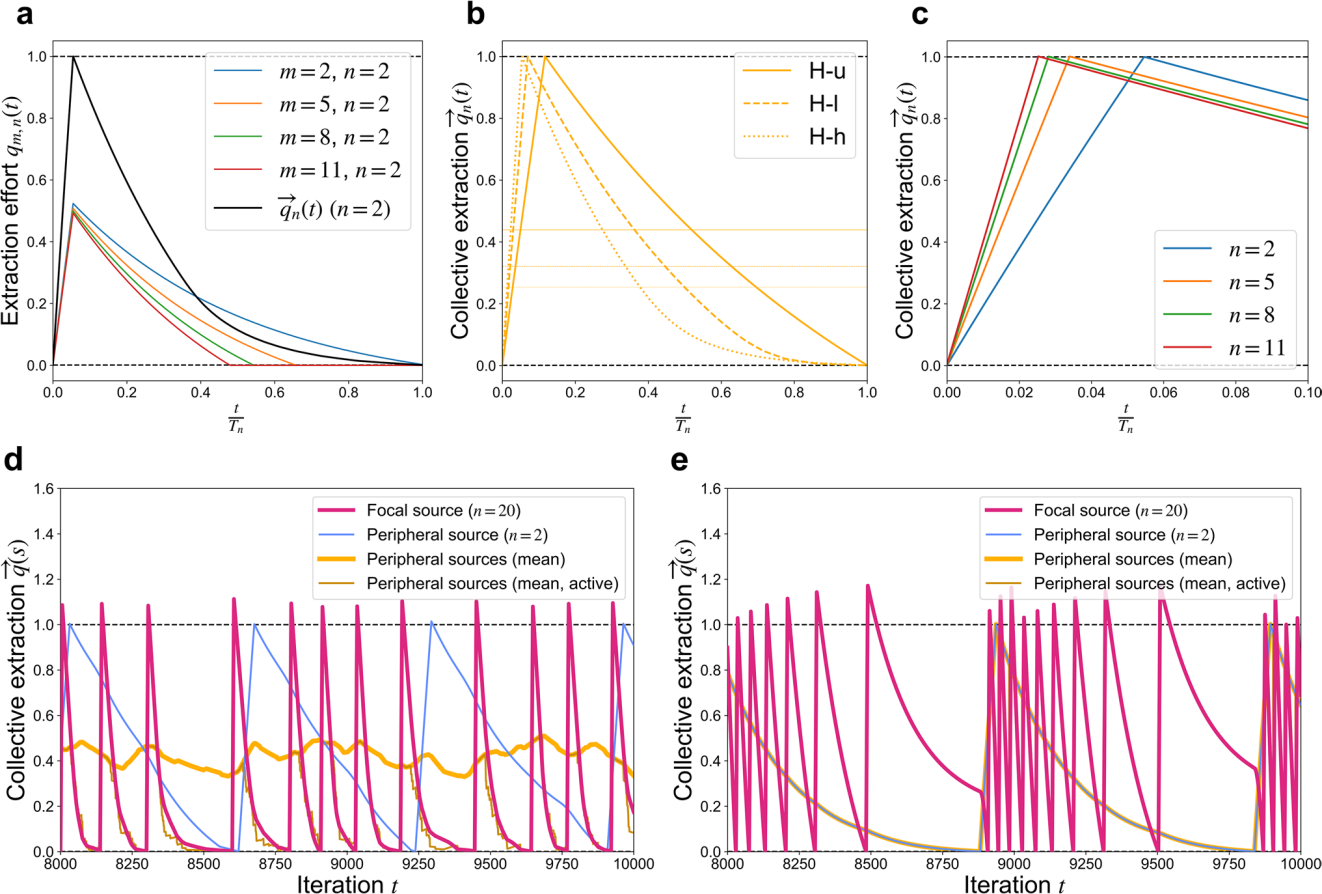
(**a**) *Predicted time evolution of extraction levels* $q(a,s)$ through a depletion-remediation cycle for agents of various degrees $m(a)$ from sources with degree $n(s)=2$ within L–h networks based on a heterogeneous mean-field approach. (**b**) *Effects of agent degree heterogeneity*: Predicted time evolution of collective extraction $\overrightarrow{q}(s)$ with different types of agent degree heterogeneity (u, l, h) for sources with degree $n(s)=2$ within H networks; (**c**) *Role of source degree*: Predicted time evolution of collective extraction $\overrightarrow{q}(s)$ for sources of various degree $n(s)$ within L-l networks. (**d**) *Dynamics of collective extraction* from simulation on star network with randomized initial conditions ($u=1$, $k=.01$; “Active” indicates that peripheral-source extraction values are set to zero for “inactive” agents whose current extraction from the focal source is zero). (**e**) *Dynamics of collective extraction* from simulation on star network with initial conditions synchronized such that all sources have the same collective extraction, providing an exaggerated illustration of how lower-degree sources can slow the remediation times of higher-degree sources.

Simulation results show that degree heterogeneity among agents plays a dominant role in determining the collective wealth that a population achieves under free adaptation. Higher agent degree heterogeneity is associated with both lower extraction and lower resource quality for sources of all degrees (Fig. 2d and e), as well as reduced payoffs for most agents (Fig. 2c). This observation immediately distinguishes this bistable CPR model from previously-studied models of linearly degrading CPRs, on which agent degree heterogeneity was found to play only a minor role in determining population-level extraction and wealth at equilibrium^16^. This occurs because agents that have access to a greater number of CPRs tend to sustain higher overall extraction levels $\overleftarrow{q}\left(a\right)$. Given the increasing marginal costs associated with higher extraction, this motivates higher-degree agents to reduce their extraction more rapidly from depleted sources (Eq. 5). When a source is shared by agents that differ from one another in degree, the higher-degree agents reduce their extraction more rapidly, often reaching zero extraction as lower-degree agents gradually reduce their extraction until remediation is triggered. This is visible in curves representing the predicted time evolution of extraction levels (Fig. 3a), which we estimate here using a heterogeneous mean-field approach (see Supplementary Information S3 for details and comparisons of the model’s predictions to simulation results). This leads to the more tapered decay curves of collective extraction observed in networks with greater agent degree heterogeneity (Fig. 3b). Sources spend a greater fraction of their time in depleted states, and so exhibit reduced quality on average. Since higher-degree agents reduce extraction more rapidly, and so waste less effort attempting to extract from depleted sources. In this sense, higher-degree agents manage to overcome diminishing marginal utilities: they extract more, and yet they attain not only greater overall payoffs, but also greater payoffs *per unit extraction effort*.

### Higher-degree sources sustain lower extraction *and* lower quality under free adaptation

Higher-degree sources tend to sustain both lower average extraction and lower average quality than do lower-degree sources within the same network (Fig. 2d and e). At first, this positive correlation between extraction and resource quality may seem to contradict the definition of common-pool resources as *rivalrous*: that is, increased extraction reduces quality. This provides another sharp distinction between networks with linearly degrading CPRs and the current bistable CPR model, in which CPRs undergo sudden regime shifts in quality, and agents react in time by adjusting their extraction based on ever-changing costs and incentives. The predicted depletion-remediation cycle waveforms of collective extraction for sources of different degree within the same network type (Fig. 3c) illustrate that resource depletion is delayed at lower-degree sources. For lower-degree sources, collective extraction $\overrightarrow{q}\left(s\right)$ is coupled more strongly to the individual extraction $\overleftarrow{q}\left(a\right)$ of each of its users. For example, as the collective extraction on a source of degree *n* approaches the source’s depletion threshold ${\overrightarrow{q}}_{\mathrm{D}}$, then each of its users will apply an extraction effort approaching ${\overrightarrow{q}}_{\mathrm{D}}/n$, on average. Regardless of how these user’s other extraction levels fluctuate, their overall individual extraction values $\overleftarrow{q}\left(a\right)$ will all tend to be elevated by about ${\overrightarrow{q}}_{\mathrm{D}}/n$—that is, moreso for lower degree $n$—whenever source extraction nears depletion levels. Due to the increasing marginal costs of extraction, these elevated extraction levels will motivate agents to slow their run on the source (Eq. 5). This delays depletion, allowing the source to spend a greater fraction of its time in a viable state, and so improves average quality.

### Source degree heterogeneity is associated with reduced extraction and resource quality under free adaptation

As observed above, lower-degree sources tend to sustain higher quality than do higher-degree sources within the same network. However, this does *not* imply that networks with a greater abundance of low-degree sources (e.g., **H**igh-heterogeneity) sustain higher overall quality than do networks having fewer low-degree sources (e.g., **U**niform-degree). Instead, networks with higher heterogeneity among source degrees tend to sustain both *lower* overall extraction (Fig. 2d) and *lower* resource quality (Fig. 2e). The dynamics of extraction from one resource cannot be fully understood by only considering effects at the most local level, but rather are influenced by interactions that occur over higher-level network scales. The extraction and quality levels sustained by a source depends on its wider network context, in which extraction from one source is coupled to that at other sources through their shared agents. The effects of degree heterogeneity upon this coupling can be understood by examining how free adaptation plays out on “star” networks consisting of a high-degree source situated among several lower-degree peripheral sources (Supplementary Information S4.2). The higher the degree of a star network’s central source, the lower the collective extraction and quality sustained by *all* sources. When agents share access to a higher-degree source, then on average their individual extraction values $\overleftarrow{q}\left(a\right)$ tend to be reduced, both because lower-degree source supports lower collective extraction over time, and because each individual user is responsible for a smaller relative share of that collective extraction. The lower marginal costs associated with this reduced extraction (Eq. 5) lead to faster depletion and slower remediation—that is, reduced average quality—at these agents’ lower-degree peripheral sources. The presence of a high-degree source thus tends to drag down the quality of lower-degree sources.

Conversely, we observe interactions involving low-degree sources further dragging down the quality of higher-degree sources. The time taken for a depleted source to become remediated has a highly convex dependence on its agents’ extraction levels from other sources (Supplementary Information S4). When extraction levels at several sources are simultaneously low (Fig, 3d and e), then the reduced individual extraction levels $\overleftarrow{q}\left(a\right)$ can lead to drastically delayed remediation—and thus reduced quality—at the sources that their agents share. These coupling effects are enhanced in the presence of abundant low-degree agents, for whom each individual CPR accounts for a greater relative fraction of its total extraction $\overleftarrow{q}\left(a\right)$. Meanwhile, very high degree agents present in high-heterogeneity (**h**) networks rapidly reduce their extraction to zero at depleted sources, further enhancing this effect, particularly at higher-degree sources that are more likely to have high-degree affiliates. This is consistent with the observation of greatly increased periods of oscillation, which further inspection reveals to be due to increased depleted-state times, on **L–h** and **H–h** networks (Fig. 2f; Supplementary Information S4.3).

We note that the above analyses depend on the assumption that all sources share similar depletion and remediation thresholds (${\overrightarrow{q}}_{\mathrm{D}}$ and ${\overrightarrow{q}}_{\mathrm{R}}$) regardless of their degrees. If this were not the case, and instead—to consider an opposite extreme—these threshold values were proportional to a source’s degree, then the role of source degree heterogeneity in shaping population-level extraction patterns could be greatly reduced (Supplementary Materials S3.4.2).

### Preference for uniform adaptation improves outcomes on degree-heterogeneous networks

**Figure 4:**
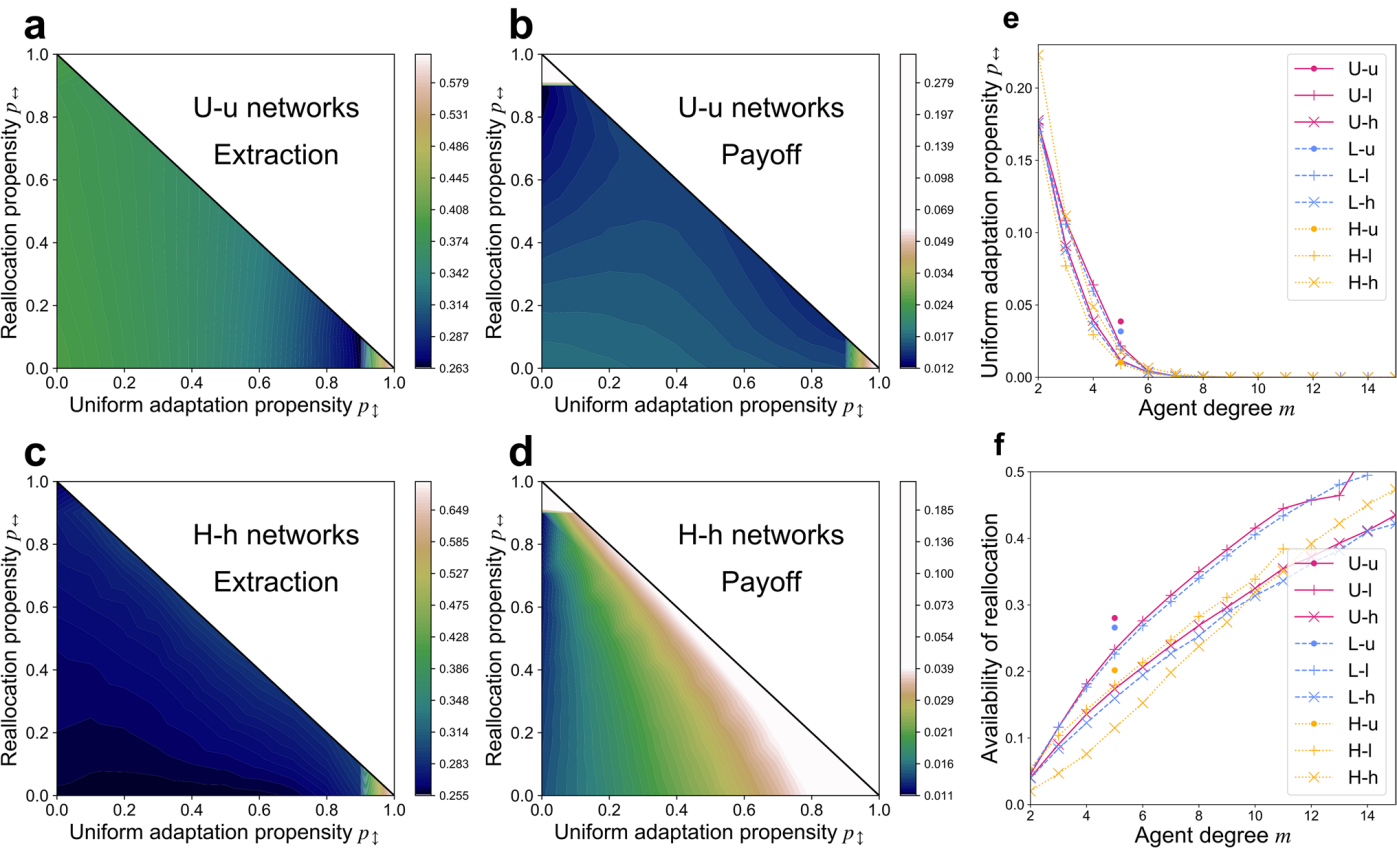
*Mixed adaptation strategies.* Contour plots interpolating mean values from simulations on ensembles of 30 networks, with data points at increments of .1 in ${p}_{\updownarrow }$ and ${p}_{\leftrightarrow }$, with ${{p}_{0}=1-p}_{\updownarrow }-{p}_{\leftrightarrow }$ (The origin (${(\mathrm{p}}_{\updownarrow },{p}_{\leftrightarrow })=(\mathrm{0,0})$) represents pure *free adaptation*, the rightmost corner ${(\mathrm{p}}_{\updownarrow },{p}_{\leftrightarrow })=(\mathrm{1,0})$ represents pure *uniform adaptation*, and the upper corner ${(\mathrm{p}}_{\updownarrow },{p}_{\leftrightarrow })=(\mathrm{0,1})$ represents pure *reallocation*): (**a**) *Extraction effort* from uniform-degree (U-u) networks; (**b**) *Payoffs* from uniform-degree (U-u) networks; (**c**) *Extraction effort* from high degree-heterogeneity (H–h) networks; and (**d**) *Payoffs* from high degree heterogeneity (H–h) networks. *Reinforcement learning:* Means over ensembles of 300 networks of each type of (**e**) *Uniform adaptation propensity* by agent degree, and (**f**) *Availability of reallocation* (the fraction of iterations in which an agent finds itself in a mixed-quality environment containing at least one each of viable and depleted sources).

Moving beyond the pure *free adaptation* case considered above, we now consider games in which all members of a population share the same mixed strategy, $({p}_{0},{p}_{\updownarrow },{p}_{\leftrightarrow })$, throughout the course of a simulation. On degree-heterogeneous (**U**-**u**) networks, populations tend to achieve a local maximum of collective wealth around pure free adaptation (${p}_{0}=1, {p}_{\updownarrow }={p}_{\leftrightarrow }=0;$ Fig. 4b); any tendency to practice uniform adaptation or reallocation instead leads to reduced extraction (Fig. 4a) and reduced payoffs (Fig. 4b). But on degree-heterogeneous (e.g., **H**–**h**) networks, as the preference for uniform adaptation is increased (${p}_{\updownarrow }\to 1$), populations steadily reduce their collective extraction (Fig. 4c) such that they sustain steadily increasing resource quality and greater payoffs (Fig. 4d).

Unlike under free adaptation, agents practicing *uniform adaptation* tend to settle into steady states (Supplementary Information S2.2). Agents stop adapting whenever they find themselves extracting at levels in which the marginal costs associated with a uniform adaptation update balance out any additional marginal payoffs the update would provide given the agent’s particular mixed-quality environment of viable and depleted sources (Eq. 6). In this way, the incorporation of uniform adaptation into agents’ adaptation strategies can disrupt the cyclical extraction dynamics that characterize pure free adaptation. As a result of each agent applying equal extraction effort across each of its links, collective extraction tends to fall upon sources proportional to their degree. Higher-degree sources thus tend to become—and remain—depleted, while lower-degree sources tend to remain viable in networks where sources vary in degree. The sustained presence of a depleted high-degree source serves to increase the mean resource depletion level $\overline{\chi }$ to which its many affiliated agents are exposed, motivating them to extract at reduced levels (Eq. 6). These reduced extraction levels in turn increase the probability that more-abundant, lower-degree sources will remain viable as dynamics stagnate. This moderating effect is enhanced in populations with an abundance of lower-degree agents, for whom these depleted high-degree sources play a relatively larger role ($\sim 1/m$) in determining individual extraction levels $\overleftarrow{q}\left(a\right)$. Agent degree heterogeneity thus enhances the effect which results from source degree heterogeneity under uniform adaptation, further increasing collective wealth. This pattern, like those discussed previously, is a unique result of the bistable behavior of the model’s CPRs, with previous results suggesting that the result would be reversed on networks of linearly degrading CPRs:  there, reduced emphasis on allocation leads to lower collective wealth than does free adaptation, with greater degree heterogeneity widening the discrepancy^16^.

Turning to reallocation, the most remarkable feature visible here is the sharp increase in payoffs as strategies approach pure reallocation (${p}_{0}=1$). Since reallocation conserves each agent’s total individual extraction in time, this result strongly reflects the initial conditions chosen. Here, extraction levels $\overleftarrow{q}\left(a\right)$ were initialized so that the total extraction effort exerted by each agent over the course of a simulation are identical in both the pure free adaptation and pure reallocation cases. While reallocation can sometimes relieve extraction from a depleted source by an amount sufficient to trigger its remediation, dynamics typically stagnate following these initial adjustments (Supplementary Materials S2.3). The superior collective wealth achieved by way of pure *reallocation* here demonstrates that if agents were to simply hold their extraction levels steady rather than seeking short-term payoff gains, then resource quality could be conserved in time. The same total effort, spread more evenly over time, would then yield much higher payoffs.

### Lower-degree agents are less prone to “unlearn” preferences for uniform adaptation or reallocation

When agents’ adaptation strategies are themselves allowed to co-evolve with extraction levels by way of reinforcement learning, the lowest-degree agents ($m=2$) sustain the highest propensities for uniform adaptation ($\overline{{p}_{\updownarrow }}\approx .17$; Fig. 4e) and reallocation ($\overline{{p}_{\leftrightarrow }}\approx .25$; Supplementary Information 2.4). Agents’ propensities for these constrained adaptation strategies then decrease steadily as their degrees increase, reaching negligibly small levels for agents with above-average degrees ($m>6$). This reflects the fact that the distinctions between these adaptation strategies are often moot for lower-degree agents, who often achieve similar payoffs regardless of their choice of adaptation strategy. Unlike higher-degree agents, lower-degree agents very rarely find themselves in mixed environments with both viable and depleted sources (Fig. 4f). As a result, reallocation from a depleted source to a viable source is not actually possible most of the time for them. Furthermore, whenever reallocation is impossible, uniform adaptation becomes equivalent to free adaptation (Eqs. 5 and 6). For higher-degree agents, however, the flexibility of free adaptation offers more substantial advantages, leading to stronger reinforcement over the other strategies. If these experiments are repeated, but with the option of free adaptation now removed from consideration by agents (${p}_{0}\equiv 0,{p}_{\updownarrow }+{p}_{\leftrightarrow }=1)$, then agents’ reallocation propensity tends to increase asymptotically with agent degree (Supplementary Information S2.4).

## Discussion

Previous social science research has sought to clarify whether common-pool resource users, faced with multiple resources and changing conditions, tend to adapt by *reallocating* their efforts, or by increasing or decreasing extraction from *all* sources. Meanwhile, many of the results reported throughout the network evolutionary game theory literature hinge upon an assumption that one aspect of individual choice—either *magnitude* or *allocation*—remains fixed. Motivated by these issues, the results presented here offer some evolutionary insights into how the structures of networked socio-ecological systems might support the emergence of preferences that effectively fix either the magnitude or allocation aspect of individual choice. Here, if individuals are inclined to practice uniform adaptation—adjusting their extraction levels at all resources by the same amount, rather than handling each separately—then they can sometimes achieve greater collective wealth than under free adaptation. Whether or not this more constrained adaptation strategy offers some advantage, though, depends strongly on the degree heterogeneity of the networked commons at hand. On degree-heterogeneous networks, introducing a preference for uniform adaptation into a population can increase resource quality and collective wealth. By allowing adaptation strategies to then co-evolve with extraction levels under reinforcement learning, we demonstrated that these constrained adaptation strategies can persist indefinitely within a population alongside free adaptation, but only among lower-degree agents. If CPR users are somehow predisposed to practice fixed-allocation (uniform) or fixed-magnitude (reallocation) adaptation, then users with access to fewer distinct resources are presented with fewer incentives to “unlearn” them.

This last result stops short of demonstrating a mechanism by which these more constrained strategies could *gain* favor, or be actively spread, throughout a community. However, since the current model has so far made no attempt to model the differences in the effective costs that would likely be associated with each adaptation strategy, this result prompts further questions. In more realistic situations, the information available to individuals may be imperfect or incomplete; adaptation strategies based on lower resolution, coarser-grained information might then emerge out of necessity. Some individuals may find it prohibitively costly to gather and manage—let alone to effectively act upon—information about too many distinct resources. Constrained adaptation strategies, resembling the uniform adaptation or reallocation strategies modelled here, might then offer additional advantages in the form of reduced cognitive or logistical costs^44,45^. Extended models that go further to account for these additional costs and advantages could potentially demonstrate how this constrained behavior—beyond merely “surviving”—might become ubiquitous within a structured population, even among higher-degree agents.

Without claiming that the model’s features apply in a straightforward way to a particular real-world commons, we nonetheless believe that these results could shed light onto actual human–environment systems. For example, our results demonstrate that differences in how the *rivalrous* property of CPRs is manifest—for instance, whether degradation is felt gradually as extraction levels increase, or instead resources undergo sudden regime changes—can lead to drastically different conclusions about how structural inequities of access shape collective behavior. This recalls the concept of “shifting baselines”^46^, originally introduced in discussions of common-pool fisheries, which highlights the implications for CPR users’ responses when environmental changes occur gradually rather than suddenly. Further experimental research has explored the differences in collective responses to gradual versus sudden changes in resource conditions^47^. More broadly, the behavior changes made in response to environmental degradation are shown to be highly dependent on time scale: sudden-onset catastrophes evoke much different adaptive responses than does gradual environmental degradation^48^. Researchers discuss the effects of “creeping normality” of slow-onset crises as affecting a society’s risk perception and collective responses^49,50^, and discourse on environmental degradation in popular culture often invokes the fable of a “boiling frog” that fails to notice gradual changes in temperature that would have evoked a violent reaction had the change been more abrupt^51,52^. These ongoing discussions in the CPR and ecology literature emphasize that these different types of resource depletion behavior can evoke drastically different adaptive responses in individual CPR users. The current model demonstrates that these different responses by individuals can lead to qualitatively distinct emergent results at the population level in networked commons.

Although the agents within this (or any) computational model may not completely capture realistic human behavior, its qualitative findings nonetheless echo some key themes that emerge in the study of real-world commons. The myopic, gain-seeking agents of the current model drive resources, one after another, to depletion, reducing their extraction only when doing so appears to be the most immediately profitable action. Even then, when we do observe that resources with fewer users sustain higher quality, we find that this occurs precisely *because* with those resources, each user assumes a greater relative share of responsibility for the resource’s quality. In networks where uniform adaptation offers an advantage, it happens precisely *because* their structures continually expose many agents to over-exploited CPRs, prompting them to moderate their overall extraction at other sources and so preserve resource quality. Each of these findings emphasize that the structures of access that help guide populations towards more optimal outcomes are typically not those that *prevent* individuals from extracting by excluding them from access to resources. Rather, structures that *expose* CPR appropriators more directly to the consequences of their actions present them with clearer incentives to moderate their extraction, and so to avert over-exploitation. In this sense, even the self-interested, short-sighted agents of this computational model—without ever encountering any fixed rule for “cooperation”—manage to demonstrate some key themes that emerge in the commons of the real world. In real-world commons, too, it seems that structures of access could also play an important role in the development of adaptation behavior that serves to reconcile individual self-interest with the longer-term, collective good.

## Supplementary Information

Supplementary Information.

## References

[1] Ostrom E (1990). Governing the Commons: The Evolution of Institutions for Collective Action.

[2] Ostrom E, Gardner R, Walker J (1994). Rules, Games, and Common-Pool Resources.

[3] Schnier KE (2009). Spatial externalities and the common-pool resource mechanism. J. Econ. Behav. Organ..

[4] Cardenas, J.-C., Janssen, M. & Bousquet, F. Dynamics of rules and resources: Three new field experiments on water, forests and fisheries. in *Handbook on experimental economics and the environment* (eds. List, J. A. & Price, M. K.) 319–345 (Edward Elgar, 2013).

[5] Prediger S, Vollan B, Frölich M (2011). The impact of culture and ecology on cooperation in a common-pool resource experiment. Ecol. Econ..

[6] Castillo D, Bousquet F, Janssen MA, Worrapimphong K, Cardenas JC (2011). Context matters to explain field experiments: Results from Colombian and Thai fishing villages. Ecol. Econ..

[7] Salcedo, R. Dynamic decision making in common-pool resource economic experiments: Behavioral heterogeneity in the field and the lab. LACEEP Working Paper No. 201465 (Latin American and Caribbean Environmental Economics Program, 2014).

[8] Gehrig S, Schlüter A, Hammerstein P (2019). Sociocultural heterogeneity in a common pool resource dilemma. PLoS ONE.

[9] Weitz JS, Eksin C, Paarporn K, Brown SP, Ratcliff WC (2016). An oscillating tragedy of the commons in replicator dynamics with game-environment feedback. Proc. Natl. Acad. Sci..

[10] Ricoveri, G. *Nature for Sale: The Commons versus Commodities*. (Pluto Press, 2015). doi:10.2307/j.ctt183p2rv.

[11] Cerutti N (2017). Effects of space in a dynamic common-pool resource experiment. SSRN Electron. J..

[12] Barnes M, Kalberg K, Pan M, Leung P (2016). When is brokerage negatively associated with economic benefits? Ethnic diversity, competition, and common-pool resources. Soc. Netw..

[13] Farahbakhsh I, Bauch CT, Anand M (2021). Best response dynamics improve sustainability and equity outcomes in common-pool resources problems, compared to imitation dynamics. J. Theor. Biol..

[14] Sugiarto HS (2017). Social cooperation and disharmony in communities mediated through common pool resource exploitation. Phys. Rev. Lett..

[15] İlkılıç R (2011). Networks of common property resources. Econ. Theory.

[16] Schauf A, Oh P (2021). Myopic reallocation of extraction improves collective outcomes in networked common-pool resource games. Sci. Rep..

[17] Santos FC, Santos MD, Pacheco JM (2008). Social diversity promotes the emergence of cooperation in public goods games. Nature.

[18] Perc M, Gómez-Gardeñes J, Szolnoki A, Floría LM, Moreno Y (2013). Evolutionary dynamics of group interactions on structured populations: A review. J. R. Soc. Interface.

[19] Cao X-B, Du W-B, Rong Z-H (2010). The evolutionary public goods game on scale-free networks with heterogeneous investment. Phys. Stat. Mech. Appl..

[20] Li J, Wu T, Zeng G, Wang L (2012). Selective investment promotes cooperation in public goods game. Phys. Stat. Mech. Appl..

[21] Zhang H, Shi D, Liu R, Wang B (2012). Dynamic allocation of investments promotes cooperation in spatial public goods game. Phys. Stat. Mech. Appl..

[22] Wang Q (2018). Heterogeneous investments promote cooperation in evolutionary public goods games. Phys. Stat. Mech. Appl..

[23] Szolnoki A, Chen X (2020). Blocking defector invasion by focusing on the most successful partner. Appl. Math. Comput..

[24] Lee H-W, Cleveland C, Szolnoki A (2021). Small fraction of selective cooperators can elevate general wellbeing significantly. Phys. Stat. Mech. Appl..

[25] Gracia-Lazaro C (2012). Heterogeneous networks do not promote cooperation when humans play a Prisoner’s Dilemma. Proc. Natl. Acad. Sci..

[26] May RM (1977). Thresholds and breakpoints in ecosystems with a multiplicity of stable states. Nature.

[27] Scheffer M, Carpenter S, Foley JA, Folke C, Walker B (2001). Catastrophic shifts in ecosystems. Nature.

[28] Scheffer M, Carpenter SR (2003). Catastrophic regime shifts in ecosystems: Linking theory to observation. Trends Ecol. Evol..

[29] Meron, E., Mau, Y. & Zelnik, Y. R. Multistability in Ecosystems: Concerns and Opportunities for Ecosystem Function in Variable Environments. in *Mathematics of Planet Earth* (eds. Kaper, H. G. & Roberts, F. S.) vol. 5 177–202 (Springer International Publishing, 2019).

[30] Scheffer M, Carpenter S, Young B (2005). Cascading effects of overfishing marine systems. Trends Ecol. Evol..

[31] Daskalov GM, Grishin AN, Rodionov S, Mihneva V (2007). Trophic cascades triggered by overfishing reveal possible mechanisms of ecosystem regime shifts. Proc. Natl. Acad. Sci..

[32] Scheffer M, Jeppesen E (2007). Regime shifts in shallow lakes. Ecosystems.

[33] Coutinho RM, Kraenkel RA, Prado PI (2015). Catastrophic regime shift in water reservoirs and São Paulo water supply crisis. PLoS ONE.

[34] Gunderson L (2017). Regime shifts and panarchies in regional scale social-ecological water systems. Ecol. Soc..

[35] Rietkerk M (2004). Self-organized patchiness and catastrophic shifts in ecosystems. Science.

[36] Pacheco JM, Santos FC, Souza MO, Skyrms B (2009). Evolutionary dynamics of collective action in *N*-person stag hunt dilemmas. Proc. R. Soc. B Biol. Sci..

[37] Ostrom E, Janssen MA, Anderies JM (2007). Going beyond panaceas. Proc. Natl. Acad. Sci..

[38] Lahkar R, Seymour RM (2014). The dynamics of generalized reinforcement learning. J. Econ. Theory.

[39] Ohkubo J, Tanaka K, Horiguchi T (2005). Generation of complex bipartite graphs by using a preferential rewiring process. Phys. Rev. E.

[40] Baland J-M, Platteau JP (1996). Halting Degradation of Natural Resources: Is there a Role for Rural Communities?.

[41] Gintis H (2009). Game Theory Evolving: A Problem-Centered Introduction to Modeling Strategic Interaction.

[42] Ohtsuki H, Nowak MA (2006). The replicator equation on graphs. J. Theor. Biol..

[43] Börgers T, Sarin R (1997). Learning through reinforcement and replicator dynamics. J. Econ. Theory.

[44] Deck C, Jahedi S (2015). The effect of cognitive load on economic decision making: A survey and new experiments. Eur. Econ. Rev..

[45] Drichoutis AC, Nayga RM (2020). Economic rationality under cognitive load. Econ. J..

[46] Pauly D (2019). Vanishing Fish: Shifting Baselines and the Future of Global Fisheries.

[47] Cerutti N, Schlüter A (2019). Resource changes: Exogenous or endogenous, gradual or abrupt. Experimental evidence. Int. J. Environ. Stud..

[48] Koubi V, Stoll S, Spilker G (2016). Perceptions of environmental change and migration decisions. Clim. Change.

[49] Lang T (2010). Crisis? What crisis? The normality of the current food crisis. J. Agrar. Change.

[50] Loewenstein G, Mather J (1990). Dynamic processes in risk perception. J. Risk Uncertain..

[51] Tickell C (1990). Human effects of climate change: Excerpts from a lecture given to the Society on 26 March 1990. Geogr. J..

[52] Moore FC, Obradovich N, Lehner F, Baylis P (2019). Rapidly declining remarkability of temperature anomalies may obscure public perception of climate change. Proc. Natl. Acad. Sci..

