# Supplementary material for: Adaptation strategies and collective dynamics of extraction in networked commons of bistable resources
Source: Sci Rep. 2021 Nov 9;11:21987. doi: 10.1038/s41598-021-01314-2 (PMC8578606; doi:10.1038/s41598-021-01314-2)
Supplement: Supplementary file 1 — Supplementary Information. [file 41598_2021_1314_MOESM1_ESM.pdf]

# Adaptation strategies and collective dynamics of extraction in networked commons of bistable resources: SUPPLEMENTARY INFORMATION


**Andrew Schauf**[1,*] **and Poong Oh**[1]

[1]Nanyang Technological University, Wee Kim Wee School of Communication and Information, Singapore, 639798
[*]andrew.schauf@ntu.edu.sg


## SUMMARY


This document provides additional results and details to support the article "Adaptation strategies and collective dynamics of extraction in networked commons of bistable resources". We describe the article's methods in a more detailed manner in Section S1, and in Section S2 we present more complete simulations results, including results from mixed-strategy simulations on all network types, as well as from simulations where agent adaptation strategies evolve under generalized reinforcement learning. In Section S3, we discuss a heterogeneous mean-field (HMF) description of free adaptation dynamics in the CPR extraction game on networks of bistable CPRs. This coarse-grained perspective aids in our interpretation of simulation results and provides the representative extraction waveforms presented in the article (Fig. 3), which illustrate how source degree and network degree distributions shape the time evolution of extraction levels from sources as they oscillate through cycles of depletion and remediation. We also apply this HMF approach to investigate a scenario where sources have *degree-proportional capacity* in lieu of ensemble simulations. In Section S4, we explore the role of source degree heterogeneity in bistable CPR extraction games using bipartite star networks and further analyses of network simulations. The roles of the model's various parameters is discussed in Section S5, and the HMF approach is used to explore how changes to these parameter values could affect *free adaptation* extraction dynamics. Finally, to support some heuristic arguments made regarding the role of the typical assumption of *uniform-allocation* behavior in networked public goods games, in Section S6 we present results from a networked public goods game played without a cooperator/defector dichotomy.


## Contents


**S1 Detailed methods** **2**
S1.1 Agent-resource affiliation networks . . . 2
S1.2 CPR extraction game . . . 2
S1.3 Adaptation strategies . . . 3
Free adaptation • Uniform adaptation • Reallocation • Reinforcement learning
S1.4 Simulations . . . 5

**S2 Simulation results** **6**
S2.1 Results for mixed strategies . . . 6
S2.2 Pure uniform adaptation . . . 6
S2.3 Pure reallocation . . . 6
S2.4 Reinforcement learning . . . 6

**S3 Heterogeneous mean-field model of *free adaptation* dynamics** **6**
S3.1 Approach . . . 6
S3.2 Method 1: Analytical integration with feasibility condition relaxed . . . 13
S3.3 Method 2: Numerical integration with feasibility condition imposed . . . 14
S3.4 Results . . . 14
Uniform-capacity sources • Degree-proportional capacity sources

**S4 Role of source degree heterogeneity** **17**
S4.1 Limitations of HMF model in explaining observed role of source degree heterogeneity . . . 17
S4.2 Illustration using bipartite star networks . . . 18
S4.3 Illustration within a complex network context . . . 20

**S5 Effects of changes to parameter values 20**
S5.1 Cost parameter . . . 20
S5.2 Remediation and depletion thresholds . . . 20
S5.3 Quality parameters . . . 21
S5.4 Rate constant . . . 21
**S6 Relaxing the cooperator/defector dichotomy in networked PGGs with imitative dynamics 21**
S6.1 Role of the uniform-allocation assumption . . . 21
S6.2 Model . . . 22
S6.3 Simulation results . . . 23
**References 23**

# S1 Detailed methods

## S1.1 Agent-resource affiliation networks

We consider games involving a population $\mathbf{A} = \{a_1, \ldots, a_M\}$ of $M$ agents and an environment with $N$ sources, $\mathbf{S} = \{s_1, \ldots, s_N\}$, wherein agents' access to sources are described by bipartite graphs $g = (\mathbf{A}, \mathbf{S}, \mathbf{L})$, in which the presence of a link $(a,s) \in \mathbf{L}$ indicates that the agent $a$ has access to source $s$. We denote the set of sources affiliated with an agent $a$ as $\mathbf{S}_a = \{s \mid (a,s) \in \mathbf{L}\}$, and likewise for the population of agents affiliated with a source $s$: $\mathbf{A}_s = \{a \mid (a,s) \in \mathbf{L}\}$. We denote the network degree of an agent node $a$ (its number of affiliated sources) as $m(a) = \|\mathbf{S}_a\|$, and the degree of a source node $s$ (its number of affiliated agents) as $n(s) = \|\mathbf{A}_s\|$. We denote the minimum degrees of nodes of each type represented in a network as $n_{\min} = \min_{s\in\mathbf{S}} n(s)$ and $m_{\min} = \min_{a\in\mathbf{S}} m(a)$, and the maximum degrees of nodes of each type as $n_{\max} = \max_{s\in\mathbf{S}} n(s)$ and $m_{\max} = \max_{a\in\mathbf{S}} m(a)$. The network's distribution of agent degrees is denoted by $P_{\mathbf{A}}(m)$ and the distribution of source degrees by $P_{\mathbf{S}}(n)$. Conditional degree distributions are denoted as $P_{\mathbf{A}}(m \mid n)$ and $P_{\mathbf{S}}(n \mid m)$, respectively.

All networks were generated (using algorithms detailed in previous work that used the same network ensembles[1,2]) to share the same total number of agents $M = 50$, number of source nodes $N = 50$, and mean degrees $\langle m\rangle = \langle n\rangle = 5$, and are all restricted such that $m_{\min} = n_{\min} = 2$ for all $s \in \mathbf{S}$. Results are shown for ensembles of $10^3$ agent-resource affiliation networks each of 9 types, each representing a combination of one of three types of source degree heterogeneity (**U**niform-degree, **L**ow-heterogeneity, **H**igh-heterogeneity) with one of three similar types of agent degree heterogeneity (**u**niform-degree, **l**ow-heterogeneity, or **h**igh-heterogeneity). From the resulting ensembles, we extract degree histograms which are averaged to yield representative degree distributions $P_{\mathbf{A}}(m)$ and $P_{\mathbf{S}}(n)$ for each network type (Fig. 2a and b in the article).

## S1.2 CPR extraction game

Each agent $a$ exerts an *extraction effort* $q(a,s)$ upon each of its affiliated sources $s \in \mathbf{S}_a$. For each unit of extraction effort applied to a source $s$, the agent receives a benefit of magnitude $b(s)$ in return. The cost associated with extraction is given by a quadratic function, so that the marginal costs of extraction increase (that is, marginal utilities increase). The net *payoff*[1,3]. gathered by the agent is thus

$$f(a) = \left[\sum_{s\in\mathbf{S}_a} q(a,s)\cdot b(s)\right] - \frac{\gamma}{2}\overleftarrow{q}(a)^2, \tag{S1}$$

where $\gamma$ is a positive *cost parameter* and $\overleftarrow{q}(a)$ denotes the total extraction effort exerted by an agent $a$,

$$\overleftarrow{q}(a) = \sum_{s\in\mathbf{S}_a} q(a,s), \tag{S2}$$

which we will refer to as the agent's *individual extraction*. The *quality* of each source $s$, quantified by the benefit per unit extraction effort $b(s)$, is given by

$$b(s) = \alpha - \beta\chi(s), \tag{S3}$$

where $\alpha$ and $\beta$ are positive constants, and the $\chi(s)$ describes the source's current *state*. To model resource bistability, we allow $\chi(s)$ to occupy one of two possible states: a *viable* state $\chi(s) = 0$, characterized by source quality $b(s) = \alpha$, and a *depleted* state $\chi(s) = 1$, characterized by reduced quality $\alpha - \beta$. The state of a source depends on the total *collective extraction* effort applied by its affiliated agents,

$$\overrightarrow{q}(s) = \sum_{a\in\mathbf{A}_s} q(a,s), \tag{S4}$$

as well as its previous state. Specifically, $\chi(s)$ is given at a particular game iteration $t$ (which we indicate by subscripts where relevant) by

$$\chi_t(s) = \begin{cases} 1 & \text{, if } \chi_{t-1}(s) = 0 \text{ and } \overleftarrow{q}(s) > \overleftarrow{q}_{\mathrm{D}}(s) \\ 0 & \text{, if } \chi_{t-1}(s) = 1 \text{ and } \overleftarrow{q}(s) \leq \overleftarrow{q}_{\mathrm{R}}(s) \\ \chi_{t-1}(s) & \text{, otherwise} \end{cases}, \tag{S5}$$

where $\overleftarrow{q}_{\mathrm{D}}(s)$ denotes a source's *depletion threshold* and $\overleftarrow{q}_{\mathrm{R}}(s)$ denotes its *remediation threshold* (see Fig. 1a in the article).

## S1.3 Adaptation strategies

In an iterated CPR extraction game where agents' payoffs are given by Eq. S1 and source quality evolves according to Eqs. S3 and S5, agents adapt their extraction levels according to one of three different update rules. The first of these, *free adaptation*, represents unconstrained incremental adaptation to current resource conditions, and the additional two *constrained* update rules, *uniform adaptation* and *reallocation*, each correspond to a different type of additional constraint placed on agent's adaptation. Each agent $a$ plays a mixed strategy described by its relative probabilities of practicing each of these updates in any given game iteration: its *free adaptation propensity* $p_0(a)$, its *uniform adaptation propensity* $p_{\updownarrow}(a)$, and its *reallocation propensity* $p_{\leftrightarrow}(a)$ (so $p_0(a) + p_{\updownarrow}(a) + p_{\leftrightarrow}(a) = 1$). An agent's choice of update rule in any particular game iteration is determined by its own innate preferences; having selected an update rule, an agent adjusts its extraction levels in accord with this rule at a rate proportional to the marginal payoff it expects to attain thereby under current resource conditions. This assumption is similar to the replicator rule often used to model strategy updates in evolutionary games, whereby the rate at which agents adopt a certain strategy is proportional to the increase in payoff (i.e., fitness) that they anticipate they will gain thereby. These update rules are detailed below.

### *S1.3.1 Free adaptation*

When practicing *free adaptation*, an agent adjusts the extraction levels $q(a,s)$ at each of its affiliated sources $s \in \mathbf{S}_a$ separately at a rate proportional to the increase in payoffs $f(a)$ associated with each:

$$\frac{\mathrm{d}q(a,s)}{\mathrm{d}t} = k\frac{\partial f(a)}{\partial q(a,s)}, \tag{S6}$$

where $k$ is a positive rate constant. By Eq. S1, this marginal payoff increase is

$$\frac{\partial f(a)}{\partial q(a,s)} = \alpha - \beta\chi - \gamma\overleftarrow{q}(a), \tag{S7}$$

and so the discretized update rule associated with *free adaptation* is

$$q_{t+1}(a,s) = q_t(a,s) + k\left[\alpha - \beta\chi_t(s) - \gamma\overleftarrow{q}_t(a)\right]. \tag{S8}$$

Under the dynamics of Eq. S6, the steady-state condition $\frac{\mathrm{d}q(a,s)}{\mathrm{d}t} = 0$ is equivalent to the condition defining best-response, Nash equilibrium states: $\frac{\partial f(a)}{\partial q(a,s)} = 0$. Within the current bistable CPR model, sources undergo sudden changes in quality, which implicitly alter the payoff functions $f(a)$, and so redefine each "steady-state" before the state can actually be reached. Nonetheless, free adaptation represents equibrium-*seeking* behavior; we thus consider the time-averaged values of extraction levels and resource conditions attained under free adaptation as a baseline for comparison, analogous to best-response, Nash equilbrium extraction states of other CPR extraction games.

### *S1.3.2 Uniform adaptation*

An agent practicing *uniform adaptation* is constrained to adjust its extraction levels $q(a,s)$ at all of its affiliated sources $s \in \mathbf{S}_a$ by the same uniform amount. As in Eq. S6, we assume that its rates of adjustment are proportional to the marginal payoff associated with such a uniformly-allocated adjustment (which we denote by $\nabla_{\updownarrow} f(a)$):

$$\frac{\mathrm{d}q(a,s)}{\mathrm{d}t} = k\nabla_{\updownarrow} f(a). \tag{S9}$$

Using Eq. S7, we find $\nabla_{\updownarrow} f(a)$ is given by

$$\nabla_{\updownarrow} f(a) = \sum_{s' \in \mathbf{S}_a} \frac{1}{m(a)} \frac{\partial f(a)}{\partial q(a,s')} = \alpha - \beta\left(\frac{1}{m(a)} \sum_{s' \in \mathbf{S}_a} \chi(s')\right) - \gamma\overleftarrow{q}(a). \tag{S10}$$

The discretized update rule is then

$$q_{t+1}(a,s) = q_t(a,s) + k\left[\alpha - \beta\overline{\chi}(a) - \gamma\overleftarrow{q}(a)\right] \tag{S11}$$

where

$$\overline{\chi}(a) = \frac{1}{m(a)} \sum_{s' \in \mathbf{S}_a} \chi(s'). \tag{S12}$$

Unlike *free adaptation* under the parameter settings used in the article (see Section S5), here there is potential for extraction dynamics to stagnate when the marginal payoff that an agent associates with a uniformly-allocated adjustment (Eq. S11) is exactly negated by the marginal cost it would require, that is, when

$$\overleftarrow{q}(a) = \frac{\alpha - \beta\overline{\chi}(a)}{\gamma}. \tag{S13}$$

When all of an agent's sources share the same state ($\chi(s) = 0$ for all $s \in \mathbf{S}_a$, or $\chi(s) = 1$ for all $s \in \mathbf{S}_a$), then uniform adaptation is equivalent to free adaptation. All things being equal, this is combinatorially likely to occur more often for lower-degree agents, for whom fewer sources have to simultaneously attain the same state to achieve $\overline{\chi}(a) = 0$ or $\overline{\chi}(a) = 1$. Higher-degree agents can achieve a greater variety of distinct values for mean source state: $\overline{\chi}(a) \in \{\frac{m_1}{m(a)} \mid m_1 \in \{0,\ldots,m(a)\}\}$.

### *S1.3.3 Reallocation*

Under adaptation by reallocation, an agent shifts an increment of extraction effort from a depleted source towards a viable source while leaving its total individual extraction $\overleftarrow{q}(a)$ unchanged. Its rate of reallocation is again related to the payoff increase per unit effort shifted to a source, which we denote by $\nabla_{\leftrightarrow} f(a)$:

$$\frac{\mathrm{d}q(a,s)}{\mathrm{d}t} = k\nabla_{\leftrightarrow} f(a). \tag{S14}$$

The marginal payoff associated with an infinitesimal shift in extraction effort from a depleted to viable source is

$$\nabla_{\leftrightarrow} f(a) = \lim_{\varepsilon\to 0} \frac{\Delta f(a)}{\varepsilon} = \lim_{\varepsilon\to 0} \frac{[\alpha]\varepsilon - [\alpha-\beta]\varepsilon}{\varepsilon} = \beta. \tag{S15}$$

If $s_{\mathrm{D}}$ denotes a randomly-selected depleted source and $s_{\mathrm{V}}$ denotes a randomly-selected viable source, the associated discretized update rule is given by

$$q_{t+1}(a,s) = \begin{cases} q_t(a,s) - k\beta & \text{, if } s = s_{\mathrm{D}} \\ q_t(a,s) + k\beta & \text{, if } s = s_{\mathrm{V}} \\ q_t(a,s) & \text{, otherwise} \end{cases}. \tag{S16}$$

If the agent does not currently have at least one depleted and one viable source among its affiliated sources, then it will be unable to reallocate and so its extraction levels will remain the same. As mentioned above, this is more likely to occur for lower-degree agents.

In comparing the dynamics of Eq. S14 with the *reallocation dynamics* studied previously[1] on networks with linearly degrading CPRs (where source quality is defined by $b(s) = \alpha - \beta\overrightarrow{q}(s)$), it is important to note that these differ slightly from the *reallocation dynamics* considered in previous work. In this context, if $\nabla_{\leftrightarrow} f(a)$ denotes the sum of marginal payoffs associated with reallocations between the source $s$ and sources $s' \in \mathbf{S}_a$, then by Eq. S14 each extraction level $q(a,s)$ varies according to

$$\frac{\mathrm{d}q(a,s)}{\mathrm{d}t} = k \sum_{s' \in \mathbf{S}_a} \left(\left[b(s') - b(s)\right] + \left[\beta(s')q(a,s') - \beta(s)q(a,s)\right]\right). \tag{S17}$$

The first term in brackets in Eq. S17 represents the payoff increases associated with shifting extraction from lower- to higher-quality sources under present conditions. The second term in brackets in Eq. S17 reflects how these payoff increases will be affected by the changes in source quality caused by the reallocation move itself. Removing this second term would reproduce the *reallocation dynamics* studied in previous work[1], where agents were assumed to base their decisions upon information about current source quality *without* anticipating how their moves would subsequently affect source quality. These dynamics approach steady-states where the burden of over-extraction is distributed evenly among sources, which were proven to achieve greater collective wealth than any initial state where sources differ in quality. If agents were indeed able to anticipate the effects that their reallocations will have upon resource quality, then the full dynamics of Eq. S17 apply. Without studying the resulting dynamics in greater detail, we note that extraction dynamics would result in less optimal states than were reached by the myopic reallocation dynamics studied previously. Within the current bistable-CPR model, however, resource quality remains constant as collective extraction is varied (except when it undergoes an unexpected, discontinous transition), the distinction is moot.

### S1.3.4 Reinforcement learning

In the final subsection of the article's Results, we consider a scenario wherein agents dynamically adapt their update strategies while engaging in the iterative extraction game described above. That is, instead of being treated as constant parameters, the probabilities $p_0(a)$, $p_\updownarrow(a)$, and $p_\leftrightarrow(a)$ are allowed vary over time according to generalized reinforcement learning[4,5], rather than remaining fixed throughout the duration of the iterated game. At each game round, agents choose to enact a particular update rule with a probability given by the corresponding parameter value. If this update leads to an increased fitness, then its relative propensity to enact that update rule is reinforced by an incremental increase in the value of that parameter. For example, if an agent $a$ practices a *uniform adaptation* update and then receives payoff $f_t(a)$ such that $\Delta f_t(a) = f_t(a) - f_{t-1}(a) > 0$, then *positive reinforcement* occurs. Its adaptation strategy is adjusted by

$$p_{\updownarrow,t+1}(a) = p_{\updownarrow,t}(a) + \tau\Delta f_t(a) \cdot \left[1 - p_{\updownarrow,t}(a)\right], \tag{S18}$$

$$p_{\leftrightarrow,t+1}(a) = p_{\leftrightarrow,t}(a) - \tau\Delta f_t(a) \cdot p_{\leftrightarrow,t}, \tag{S19}$$

and

$$p_{0,t+1}(a) = p_{0,t+1}(a) - \tau\Delta f_t(a) \cdot p_{0,t}(a), \tag{S20}$$

where $\tau$ is a parameter describing the learning rate (for all simulations that apply reinforcement learning presented here, we set this to $\tau = 0.3$). If instead the agent's payoff decreases after enacting a particular update rule, then *negative reinforcement* occurs, and its relative propensity to choose that rule in the future is decreased. Again in the case of a uniform adaptation update, its update strategy would then by adjusted according to

$$p_{\updownarrow,t+1}(a) = p_{\updownarrow,t}(a) - \tau\Delta f_t(a) \cdot p_{\updownarrow,t}(a), \tag{S21}$$

$$p_{\leftrightarrow,t+1}(a) = p_{\leftrightarrow,t}(a) + \tau\Delta f_t(a) \cdot p_{\updownarrow,t}(a) \left[\frac{1 - p_{\leftrightarrow,t}(a)}{1 + p_{\updownarrow,t}(a)}\right], \tag{S22}$$

and

$$p_{0,t+1}(a) = p_{0,t}(a) + \tau\Delta f_t(a) \cdot p_{\updownarrow,t}(a) \left[\frac{1 - p_{0,t}(a)}{1 + p_{\updownarrow,t}(a)}\right]. \tag{S23}$$

Similar expressions apply for *reallocation* and *free adaptation* with the appropriate permutations of variables.

## S1.4 Simulations

Each round of an iterated game involves the following steps:

1. Agents collect payoffs based on their current extraction levels $q(a,s)$ and resource conditions $\chi(s)$ (Eq. S1)
2. Each agent is potentially selected for update with probability $u$ (setting $u < 1$ results in asynchronous updates)
3. Each updating agent $a$ randomly selects an update rule based on its adaptation strategy $\left(p_0(a), p_\updownarrow(a), p_\leftrightarrow(a)\right)$
4. Updating agents each adjust their extraction levels in accord with their their chosen update rules (Eq. S8, Eq. S11, or Eq. S16)
5. If applicable, the adaptation strategies of updating agents are adjusted by reinforcement learning (e.g., Eqs. S18–S23)
6. The states of all sources are updated based on these updated extraction levels (Eq. S5)

In all simulations presented in the main article, all sources are set to share parameter values $\alpha = \beta = 1$, with the same threshold values $\vec{q}_{\mathrm{D}} = 1$ and $\vec{q}_{\mathrm{r}} = .001$, and initial state $\chi(s) = 0$ for all $s \in \mathbf{S}$. The cost parameter is consistently set to $\gamma = 0.2$. The consequences of changing these parameter values are discussed in Section S5.

In simulations of mixed-strategy dynamics presented in Fig. 2 and Fig. 4a-d, Figs. S1–S10 and Fig. S20, agents update asynchronously with $u = .5$ and $k = .02$. Results represent means of time-averaged values on ensembles of 30 networks taken from the larger ensembles of $10^3$ networks that define the degree histograms (Fig. 2a and b) used in HMF computations. For

the results presented in Fig. 2, Fig. 4e and f, and Figs. S11–S16, means are taken over larger sub-ensembles of 300 networks. For pure *free adaptation* simulations ($p_0 = 1$), initial extraction levels were randomized such that $q_{t=0}(a,s) \in \left[0, \frac{\overrightarrow{q}_{\mathrm{D}}(s)}{n(s)}\right]$. All other simulations ($p_0 < 1$) were then initialized by setting each agent's individual extraction to its average value from the *free adaptation* simulation, ($\overleftarrow{\overline{q}\,(a)}_0$), allocated equally among its affiliated sources ($q_{t=0}(a,s) = \frac{\overleftarrow{\overline{q}\,(a)}_0}{m(a)}$). Simulations were iterated through $t_f = 10^5$ steps, and time-averaged quantities were computed over the final $8 \times 10^4$ iterations: e.g., $\overline{q}(a,s) = \frac{1}{8\times10^4}\sum_{t=2\times10^8} q_t(a,s)$. In mixed-strategy simulations where reinforcement learning is applied (Fig. 4e and f, and Fig. S9), all agents are initialized with $p_{\updownarrow} = p_{\leftrightarrow} = .333$ and $\tau = .3$; in those where *free adaptation* is excluded (Fig. S10), agents are initialized with $p_{\updownarrow} = p_{\leftrightarrow} = .5$. Simulations on star networks (Fig. 3d and e, and Fig. S19) used a synchronous update with $u = 1$ and $k = .01$, with means taken over 60 simulations on each star network, each with a different initial condition. These simulations were iterated through $10^4$ iterations, with averages computed over the final $9 \times 10^3$ iterations.

## S2 Simulation results

### S2.1 Results for mixed strategies

Figs. 4a-d in the article presented plots interpolating ensemble mean population-level extraction and collective wealth values for a range of mixed strategies $(p_0, p_{\updownarrow}, p_{\leftrightarrow})$ on two network types (**U-u** and **H-h**). Here, we include more complete results from these simulations. In Fig. S1, we present ensemble mean population-level *total extraction* values for all 9 network types. Fig. S2 similarly presents mean *resource quality*, and Fig. S3 shows total *collective wealth* values. The edges of these triangular plots represent mixed strategies where one adaptation strategy is omitted, and so illustrate how quantities vary as one adaptation strategy is gradually introduced alongside another. In Fig. S4, we compile results for the horizontal axes of these plots, where *uniform adaptation* propensity is increased atop *free adaptation*, with no reallocation ($p_{\leftrightarrow} = 0$). Fig. S5 illustrates the increasing introduction of a preference for *reallocation* alongside *free adaptation*, with no uniform adaptation practiced ($p_{\updownarrow} = 0$). Results from the diagonals of these plots, which represent combinations of *uniform adaptation* and reallocation only ($p_0 = 0$), are shown in Fig. S6.

### S2.2 Pure uniform adaptation

Ensemble mean time-averaged quantites for simulations of pure *uniform adaptation* dynamics ($p_{\updownarrow} \equiv 1$) are shown in Fig. S7, illustrating the approximately linear decrease of mean source quality as source degree increases.

### S2.3 Pure reallocation

Ensemble mean time-averaged quantites for simulations of pure *reallocation* dynamics ($p_{\leftrightarrow} \equiv 1$) are shown in Fig. S8. Initial conditions in these simulations are set such that collective pressure increases with source degree, so that higher-degree sources tend to be initially depleted. Results illustrate that reallocation moves then serve to shift extraction effort away from these higher-degree sources (Fig. S8a), sometimes successfully facilitating their remediation (Fig. S8b) before dynamics stagnate. In these mostly-viable environments, agents receive much higher payoffs (Fig. S8d) than they do under *free adaptation* with the same time-averaged extraction levels (Fig. S15c)

### S2.4 Reinforcement learning

Results from simulations wherein agents' adaptation strategies evolve by generalized reinforcement learning (as partially presented in Fig. 4e and f), are shown in Fig. S9. The lowest-degree agents retain a significant preference for constrainted adaptation strategies; these mean uniform update and reallocation propensities decrease almost linearly as agent degree increases until finally reaching near-zero values for agents with degrees above the mean degree $\langle m \rangle = 5$. These experiments were repeated, but with *free adaptation* removed from agents' adaptation strategies, such that $p_0(a) \equiv 0$ and $p_{\updownarrow}(a) + p_{\leftrightarrow}(a) \equiv 1$ (with the update rules of Eqs. S18–S23 modified accordingly). As the results in Fig. S10 show, higher-degree agents retain a preference for reallocation in this case.

## S3 Heterogeneous mean-field model of *free adaptation* dynamics

### S3.1 Approach

In the article, we aim to understand the patterns of extraction that emerge in a network CPR extraction game involving bistable resources: specifically, how extraction flows to or from a node tend to depend on the node's degree, and how that dependence is shaped by the degree distributions of the surrounding network. Rather than being focused on the details of any individual nodes within a particular network, or on specific events that occur throughout the course of the system's dynamics, we are primarily concerned with how extraction flows to or from a node tend to depend on a node's degree within a given type of network, on average, over time. Given this focus, we adopt a heterogeneous mean-field (HMF) perspective, where nodes of a common

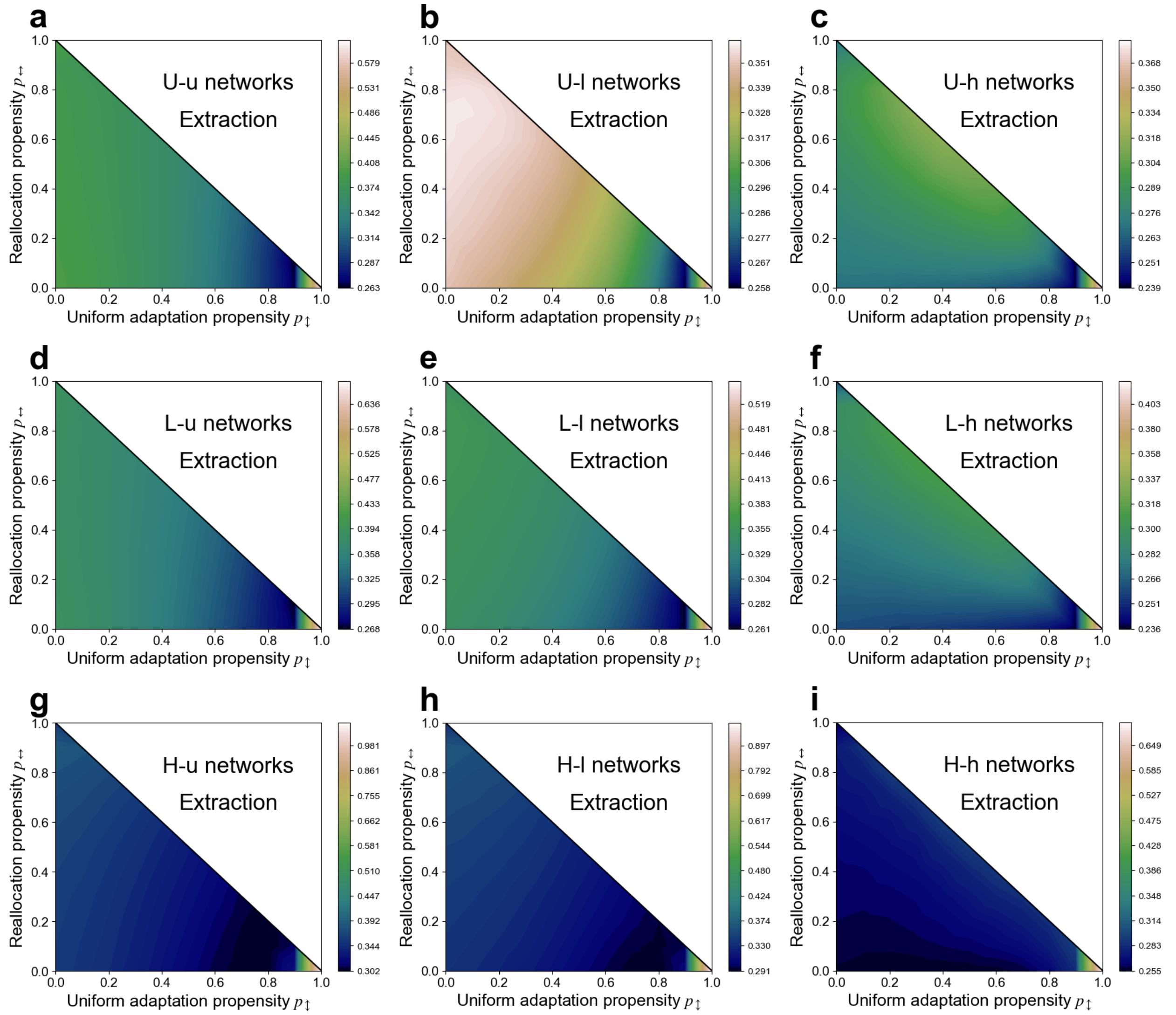


**Figure S1.** Ensemble mean time-averaged **total extraction** from ensembles of 30 networks for mixed adaptation strategies ($p_0 = 1 - p_{\updownarrow} - p_{\leftrightarrow}$) interpolated from data points at increments of .1 in $p_{\updownarrow}$ and $p_{\leftrightarrow}$, for 9 network types: **(a) U-u**, **(b) U-l**, **(c) U-h**, **(d) L-u**, **(e) L-l**, **(f) L-h**, **(g) H-u**, **(h) H-l**, and **(i) H-h**.

degree are treated as a single entity, and described in terms of their expected collective behavior, with degree distributions used to estimate the relative frequencies of interaction between nodes of different degree classes. To the extent that the model's predictions match the results of ensemble simulations, such a model can aid in our interpretations of simulation results, for example by providing dynamical equations that more explicitly include node degrees and degree distributions. Here, they also provide a representative illustration of the typical time evolution of extraction levels throughout cycles of depletion-remediation (Fig. 3a-c in the article), stripped of the "noise" of incidental fluctuations in extraction levels. The deviations of the model's predictions from simulation results can also be informative, identifying features of extraction dynamics that might be explained by "higher-order" aspects of network topology not captured by a network's degree distributions alone (see, for example, Section S4).

To derive such a model, we consider the *free adaptation* dynamics described by Eq. S8. Considering the extraction across a single link $q(a,s)$, separating out $q(a,s)$ from the other contributions to the agent's extraction intensity $\overleftarrow{q}(a)$ gives

$$\frac{\mathrm{d}}{\mathrm{d}t} q(a,s) = k \left[ \alpha - \beta \chi(s) - \gamma \left( \sum_{s' \in \mathbf{S}_a \setminus \{s\}} q(a,s') \right) - \gamma q(a,s) \right]. \tag{S24}$$

The time evolution of extraction across each link, $q(a,s)$, is coupled to that of the extraction levels across the agent's other

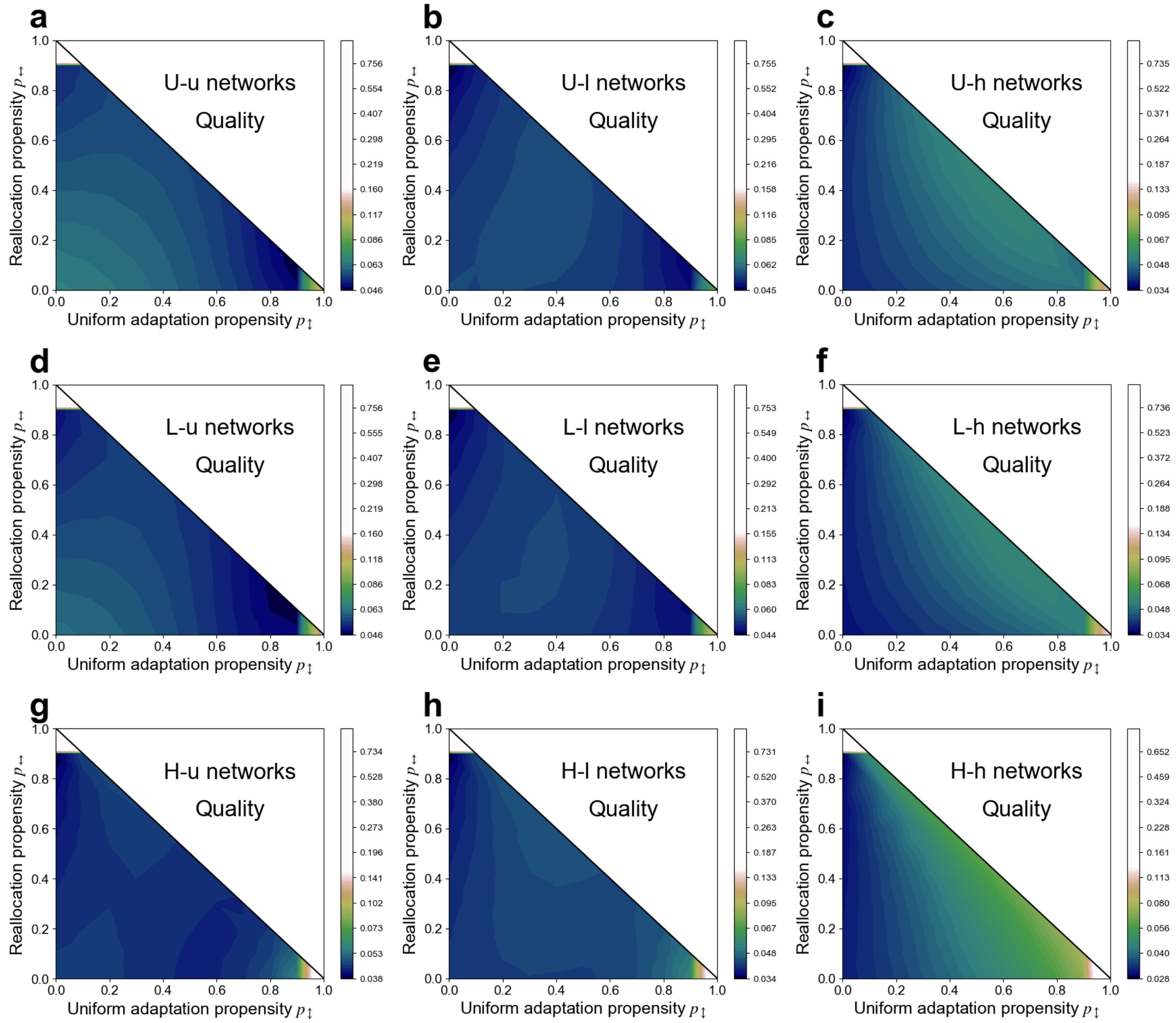


**Figure S2.** Ensemble mean time-averaged **source quality** per link from ensembles of 30 networks for mixed adaptation strategies ($p_0 = 1 - p_{\updownarrow} - p_{\leftrightarrow}$) interpolated from data points at increments of .1 in $p_{\updownarrow}$ and $p_{\leftrightarrow}$, for 9 network types: **(a) U-u**, **(b) U-l**, **(c) U-h**, **(d) L-u**, **(e) L-l**, **(f) L-h**, **(g) H-u**, **(h) H-l**, and **(i) H-h**.

links $q(a,s')$, which fluctuate independently of one another depending on the current states of each source $s'$. To describe the "typical" time evolution of extraction across a link $q(a,s)$ using Eq. S24, some estimate of the "typical" values of $q(a,s')$ is required. Here, as a first approximation, we substitute the extraction levels $q(a,s')$ by their *time-averaged* values, $\overline{q(a,s')}$.

Shifting to a heterogeneous mean-field perspective, we aim to describe the characteristic time evolution of extraction levels $q(a,s)$ based on the degrees $m(a)$ and $n(s)$ of the nodes involved: $q(a,s) \to q_{m,n}$. We thus model the expected time evolution of extraction effort across a link between a degree-$m$ agent and a degree-$n$ source[1], denoted as $q_{m,n}$ by recasting Eq. S24 as

$$\frac{\mathrm{d}}{\mathrm{d}t} q_{m,n} = k\left[\alpha - \beta\chi - \gamma(m-1)\,\overline{q}_m - \gamma q_{m,n}\right], \tag{S25}$$

where $\overline{q}_m$ denotes the expected extraction per link by a degree-$m$ agent, estimated using the time-averaged extraction values $\overline{q}_{m,n'}$:

$$\overline{q}_m = \sum_{n=1}^{n_{\max}} P_{\mathrm{S}}\left(n' \mid m\right) \cdot \overline{q}_{m,n'}. \tag{S26}$$

[1]Note that Eq. S25 shows no dependence upon the network's total number of agents $M$ or sources $N$, but rather depends upon the degrees $m$ and $n$ of the nodes involved as well as upon the network's degree distributions.

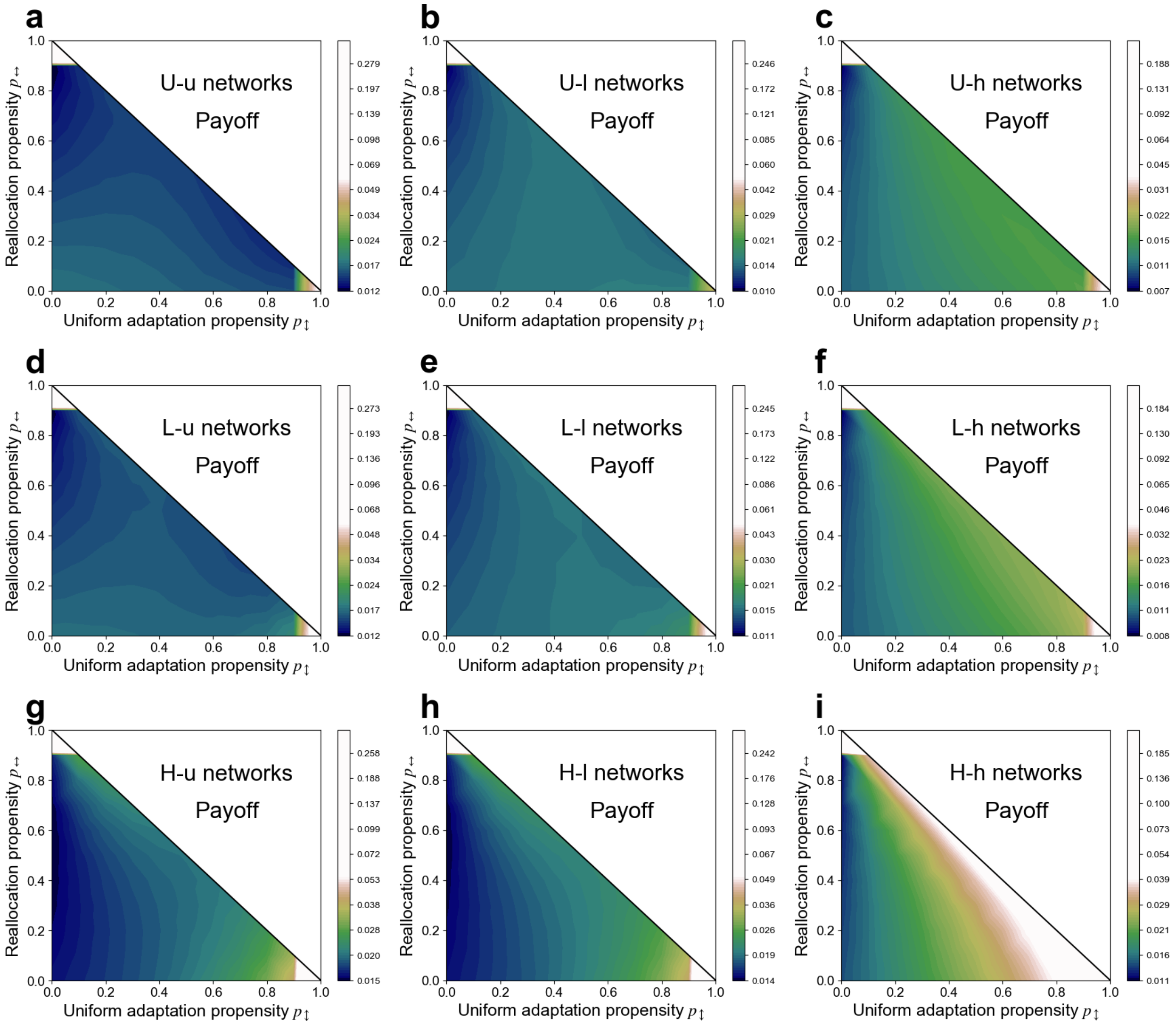


**Figure S3.** Ensemble mean time-averaged **collective wealth** (total payoff) from ensembles of 30 networks for mixed adaptation strategies ($p_0 = 1 - p_{\updownarrow} - p_{\leftrightarrow}$) interpolated from data points at increments of .1 in $p_{\updownarrow}$ and $p_{\leftrightarrow}$, for 9 network types: **(a) U-u**, **(b) U-l**, **(c) U-h**, **(d) L-u**, **(e) L-l**, **(f) L-h**, **(g) H-u**, **(h) H-l**, and **(i) H-h**.

Given a set of estimates of the time-averaged extraction effort values $\overline{q}_{m,n}$ for all degrees $m$ and $n$ represented in the system (we will denote a set of such estimates as $\overline{\mathbf{q}}$), we can thus predict the time evolution of the extraction levels $q_{m,n}(t)$ using Eq. S24, with changes in source state $\chi$ triggered as the expected extraction pressure $\overrightarrow{q}_n$,

$$\overrightarrow{q}_n = n \cdot \sum_{m'=1}^{m_{\max}} P_{\mathbf{A}}\left(m' \mid n\right) \cdot q_{m',n}(t), \tag{S27}$$

reaches thresholds $\overrightarrow{q}_{\mathrm{D},n}$ or $\overrightarrow{q}_{\mathrm{R},n}$. Below we present two different methods for determining the time evolution $q_{m,n}(t)$: first, by integrating analytically with certain constraints relaxed, and second, numerically. Once the details of the time evolution of $q_{m,n}(t)$ have been estimated (We denote as $q_{m,n}(t,\overline{\mathbf{q}})$ the estimated solution $q_{m,n}(t)$ of Eq. S25 associated with a set of estimated time-averaged extraction values $\overline{\mathbf{q}}$), then the corresponding time-averaged extraction level $\overline{q}_{m,n}$ can be computed by averaging

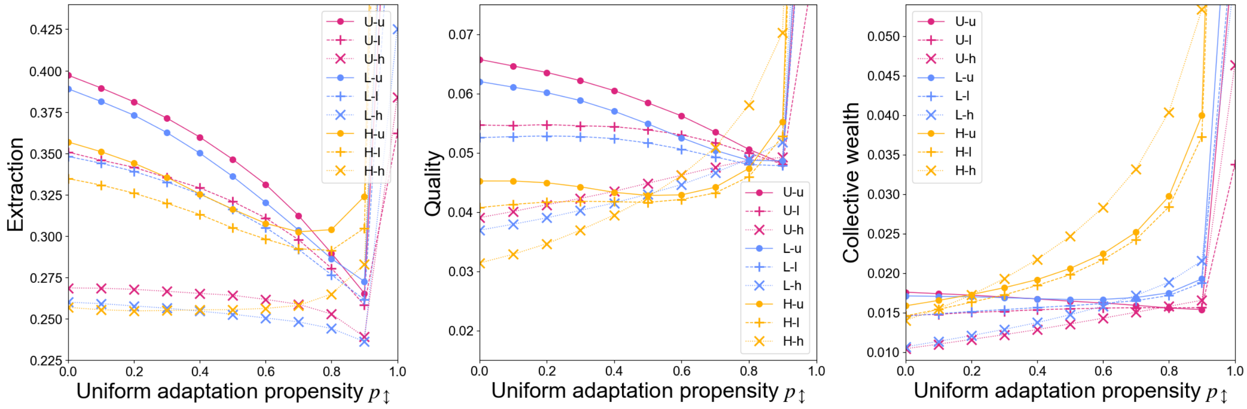


**Figure S4.** Ensemble mean time-averaged values from simulations on 30 networks each for **(a)** Total extraction, **(b)** Source quality per link, and **(c)** Collective wealth for mixed adaptation strategies of *uniform adaptation* and *free adaptation* ($p_0 = 1 - p_\updownarrow$).

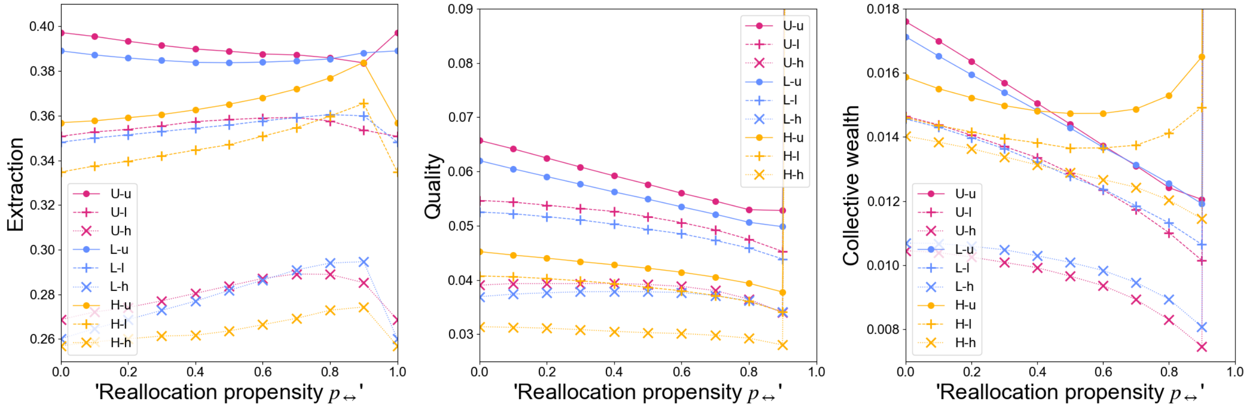


**Figure S5.** Ensemble mean time-averaged values from simulations on 30 networks each for **(a)** Total extraction, **(b)** Source quality per link, and **(c)** Collective wealth for mixed adaptation strategies of *reallocation* and *free adaptation* ($p_0 = 1 - p_\leftrightarrow$).

$q_{m,n}(t)$ over a full depletion-remediation cycle[2]:

$$\overline{q}_{m,n} = \frac{1}{[T_{\mathrm{V},n} + T_{\mathrm{D},n}]} \int_0^{T_{\mathrm{V},n}+T_{\mathrm{D},n}} q_{m,n}\left(t; \overline{\mathbf{q}}\right) \, \mathrm{d}t, \tag{S28}$$

where $T_{\mathrm{V},n}$ is the degree-$n$ source's *viable-state time* (the time elapsed between remediation and depletion events), and $T_{\mathrm{D},n}$ is its *depleted-state time* (the time elapsed between depletion and remediation events). This is circular, of course; $\overline{\mathbf{q}}$ is required to compute $\overline{\mathbf{q}}$. A set of estimated time-averaged extraction levels $\overline{\mathbf{q}}$ that could feasibly describe the time-averaged dynamics of *free adaptation* should be *self-consistent* in that the input values $\overline{\mathbf{q}}$ used to estimate each $q_{m,n}(t)$ by Eq. S25 are reproduced when these $q_{m,n}(t)$ are averaged over time in Eq. S28. Two approaches for computing self-consistent $\overline{\mathbf{q}}$ are detailed in the following sections.

Having obtained self-consistent estimates $\overline{\mathbf{q}}$, we can use these values to compute time-averaged quantities of interest: the expected *extraction pressure* $\overrightarrow{q}_n$,

$$\overrightarrow{q}_n = n \cdot \sum_{m=1}^{m_{\max}} P_{\mathbf{A}}\left(m \mid n\right) \cdot \overline{q}_{m,n}, \tag{S29}$$

[2]Here, we have assumed that sources will alternate between viable and depleted states indefinitely. For the case of complete resource depletion ($\alpha = \beta$) presented in the article, this is guaranteed to hold. However, in cases of less-drastic depletion ($\beta < \alpha$), the system's tendency to either sustain these cycles or to stagnate comes to depend upon the relative values of $\alpha$, $\beta$, and other parameters like $\gamma$ and $\overrightarrow{q}_{\mathrm{R}}$ (see Section S5 below). To simplify our analyses, we restrict our focus to the oscillatory case where depletion is always sufficiently extreme to prompt remediation under free adaptation.

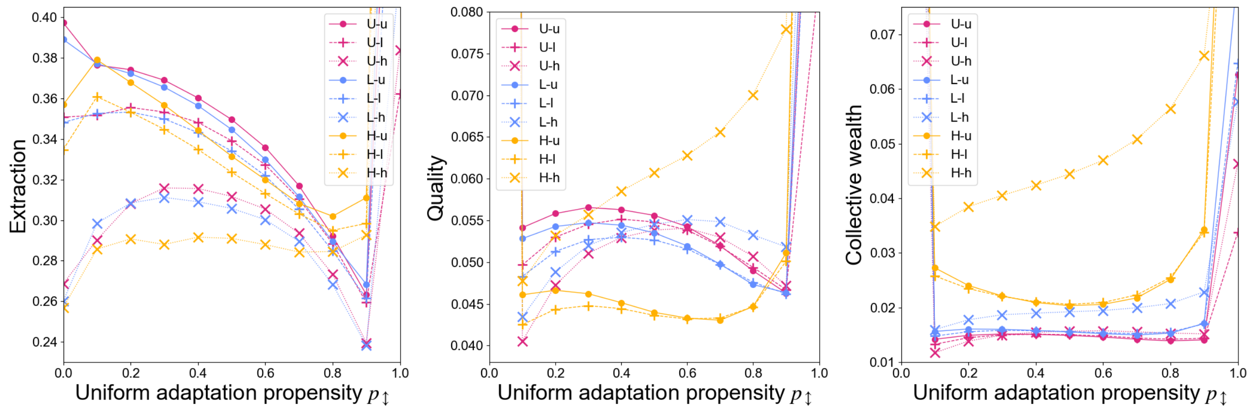


**Figure S6.** Ensemble mean time-averaged values from simulations on 30 networks each for **(a)** Total extraction, **(b)** Source quality per link, and **(c)** Collective wealth for mixed adaptation strategies of *uniform adaptation* and *reallocation* ($p_{\leftrightarrow} = 1 - p_{\updownarrow}$).

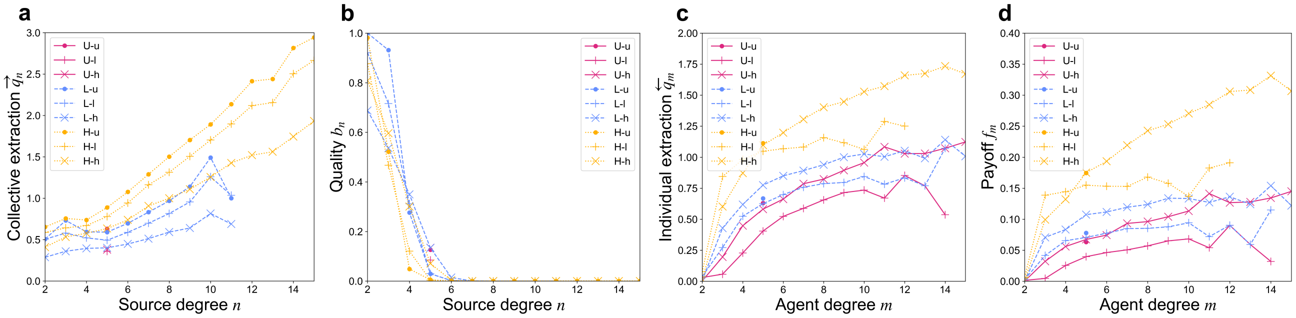


**Figure S7.** Ensemble mean time-averaged quantites from simulations on 300 networks each for *pure uniform adaptation* dynamics ($p_{\updownarrow} \equiv 1$): **(a)** Collective extraction by source degree, **(b)** Source quality by source degree, **(c)** Individual extraction by agent degree, and **(d)** Payoffs by agent degree.

and the expected *source quality* $\overline{b}_n$,

$$\overline{b}_n = \alpha - \beta \overline{\chi} = \alpha - \beta \left[ \frac{T_{\mathrm{D},n}}{T_{\mathrm{V},n} + T_{\mathrm{D},n}} \right], \tag{S30}$$

as functions of source degree $n$. Similarly, we compute expected *extraction intensity* $\overleftarrow{\overline{q}}_m$ as a function of agent degree $m$

$$\overleftarrow{\overline{q}}_m = m \cdot \sum_{n=1}^{n_{\max}} P_{\mathrm{S}}(n \mid m) \cdot \overline{q}_{m,n}. \tag{S31}$$

To compute expected agent payoffs $\overline{f}_m$, we use the time-averaged viable-state extraction levels $\overline{q_{\mathrm{V}}}_{m,n}$,

$$\overline{q_{\mathrm{V}}}_{m,n} = \frac{1}{T_{\mathrm{V},n}} \int_0^{T_{\mathrm{V},n}} q_{m,n}(t; \mathbf{q}) \, \mathrm{d}t, \tag{S32}$$

from which we can recover the depleted-state extraction levels $\overline{q_{\mathrm{D}}}_{m,n}$ using the relation

$$\overline{q}_{m,n} = \frac{T_{\mathrm{V},n} \cdot \overline{q_{\mathrm{V}}}_{m,n} + T_{\mathrm{D},n} \cdot \overline{q_{\mathrm{D}}}_{m,n}}{T_{\mathrm{V},n} + T_{\mathrm{D},n}} \tag{S33}$$

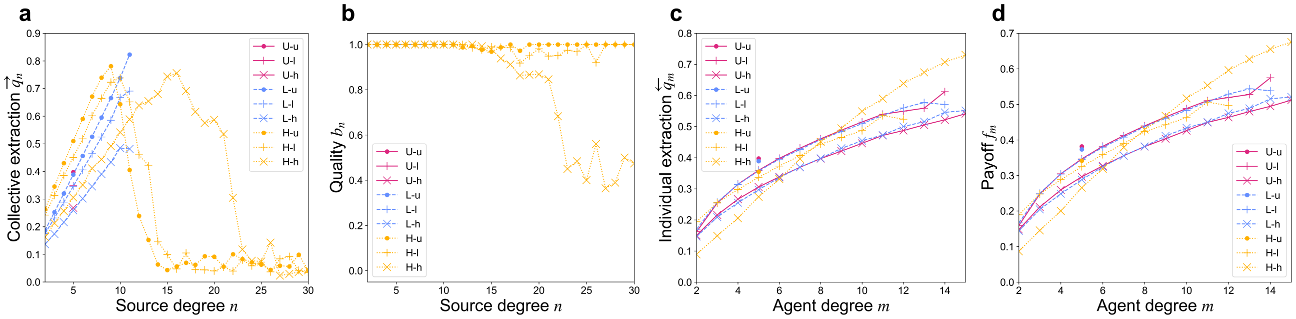


**Figure S8.** Ensemble mean time-averaged quantites from simulations on 300 networks each for *pure reallocation* dynamics ($p_{\leftrightarrow} \equiv 1$): **(a)** Collective extraction by source degree, **(b)** Source quality by source degree, **(c)** Individual extraction by agent degree, and **(d)** Payoffs by agent degree.

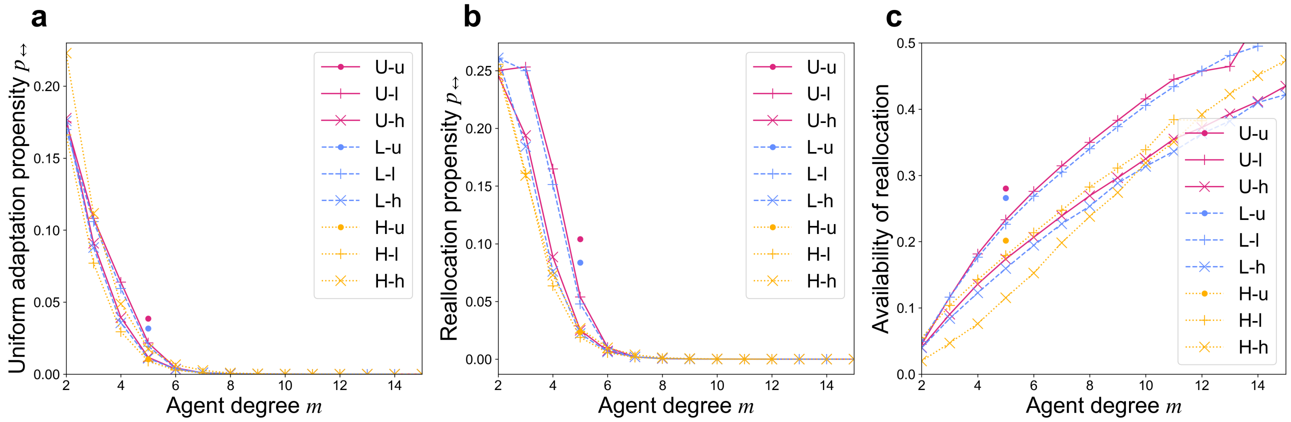


**Figure S9.** Mean steady-state adaptation strategies under generalized reinforcement learning ($p_0 = 1 - p_{\updownarrow} - p_{\leftrightarrow}$) from ensembles of 300 networks each: **(a)** Uniform update propensity $\overline{p_{\updownarrow}}$ by agent degree, **(b)** Reallocation propensity $\overline{p_{\leftrightarrow}}$ by agent degree, and **(c)** Availability of reallocation (i.e., the fraction of iterations in which an agent has at least one each of depleted and viable sources: $0 < \overline{\chi}(a) < 1$) by agent degree.

to compute expected *payoffs*

$$\overline{f}_m = m \cdot \left[ \sum_{n=1}^{n_{\max}} P_S(n \mid m) \cdot \left( \alpha \left[ \frac{T_{V,n}}{T_{V,n} + T_{D,n}} \right] \overline{q_{V\,m,n}} + (\alpha - \beta) \left[ \frac{T_{D,n}}{T_{V,n} + T_{D,n}} \right] \overline{q_{D\,m,n}} \right) \right] - \frac{\gamma}{2} \overline{\overleftarrow{q}}_m^{\,2} \qquad \text{(S34)}$$

$$= (\alpha - \beta)\, \overline{\overleftarrow{q}}_m + m \cdot \left[ \beta \sum_{n=1}^{n_{\max}} P_S(n \mid m) \cdot \left( \left[ \frac{T_{V,n}}{T_{V,n} + T_{D,n}} \right] \overline{q_{V\,m,n}} \right) \right] - \frac{\gamma}{2} \overline{\overleftarrow{q}}_m^{\,2} \qquad \text{(S35)}$$

as a function of agent degree $m$.

We now present two approaches for computing a set of self-consistent estimates $\overline{\mathbf{q}}$ using the condition given by Eqs. S25 and S28. In Method 1, we will attempt to analytically integrate Eq. S25, simplifying matters by ignoring the *feasibility condition*[3] that requires extraction levels to remain non-negative. The resulting closed-form expressions for $q_{m,n}(t)$ converts the integrals of Eq. S28 into a system of nonlinear equations, giving each $\overline{q}_{m,n}$ in terms of $\overline{\mathbf{q}}$, which can then be solved numerically. Although the resulting solutions violate this feasibility condition, their predictions nonetheless resemble simulation results in some respects, and so we present their predictions here to help clarify which aspects of the predicted extraction patterns emerge from the form of Eq. S25 alone, and which are consequences of the feasibility condition, which leads some agents to reduce their extraction to zero before others do. In Method 2, we advance Eq. S25 in time numerically while enforcing the feasibility condition. The resulting simulated trajectories $q_{m,n}(t; \overline{\mathbf{q}})$ provide an updated estimate for each $\overline{q}_{m,n}$ from Equation S28. Repeating this process iteratively, these estimates converge[4] toward a set of self-consistent estimates $\overline{\mathbf{q}}$.

[3] Again, we follow İlkılıç, who defined *feasible* states of a networked CPR extraction game as those in which all extraction levels are non-negative[3].

[4] Note that we have not rigorously addressed issues of convergence, etc., for this iterative process in general, but have observed results to converge for the

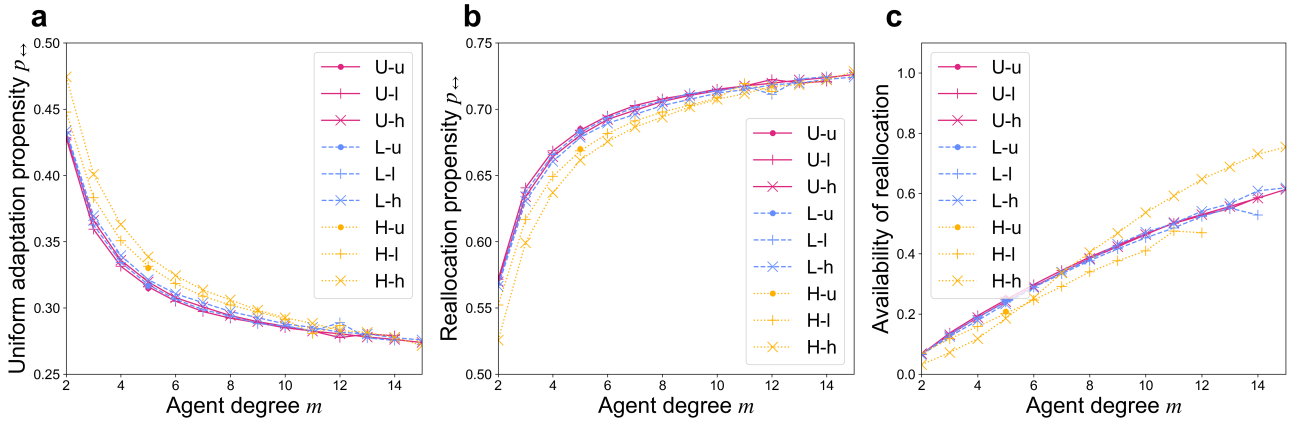


**Figure S10.** Mean steady-state adaptation strategies under generalized reinforcement learning with *free adaptation* excluded ($p_0 \equiv 0$, $p_{\updownarrow} + p_{\leftrightarrow} = 1$) from ensembles of 300 networks each: **(a)** Uniform update propensity $\overline{p_{\updownarrow}}$ by agent degree, **(b)** Reallocation propensity $\overline{p_{\leftrightarrow}}$ by agent degree, and **(c)** Availability of reallocation (i.e., the fraction of iterations in which an agent has at least one each of depleted and viable sources: $0 < \overline{\chi}(a) < 1$) by agent degree.

### S3.2 Method 1: Analytical integration with feasibility condition relaxed

To derive an expression for each $\overline{q}_{m,n}$ in terms of the other time-averaged values $\overline{q}_{m',n'}$, we first average both sides of Eq. S25 over some time interval, giving

$$\frac{\mathrm{d}}{\mathrm{d}t}\overline{q}_{m,n} = k\left[\alpha - \beta\overline{\chi}_n - \gamma(m-1)\overline{q}_m - \gamma\overline{q}_{m,n}\right]. \tag{S36}$$

If extraction dynamics have passed through some initial transient phase and then stabilized in the sense that $\overline{q}_{m,n}$ remains constant when averaged over a sufficiently long duration (as represented here by a complete depletion-remediation cycle), that is, if $\frac{\mathrm{d}}{\mathrm{d}t}\overline{q}_{m,n} = 0$[5], then Eq. S36 gives

$$\overline{q}_{m,n} = \frac{\alpha - \beta\overline{\chi}}{\gamma} - (m-1)\overline{q}_m, \tag{S37}$$

where the time-averaged state of a degree-$n$ source, $\overline{\chi}_n$, describes the fraction of each cycle that the source spends in its depleted state:

$$\overline{\chi}_n = \frac{T_{\mathrm{D},n}}{T_{\mathrm{V},n} + T_{\mathrm{D},n}}. \tag{S38}$$

To compute this viable-state time $T_{\mathrm{V},n}$ and depleted-state time $T_{\mathrm{D},n}$, we track the time evolution of the degree-$n$ source's extraction pressure $\overrightarrow{q}_n$ between thresholds by summing Eq. S25 as in Eq. S29:

$$\frac{\mathrm{d}}{\mathrm{d}t}\overrightarrow{q}_n = k\left[n\left(\alpha - \beta\chi - \gamma q'_n\right) - \gamma\overrightarrow{q}_n\right], \tag{S39}$$

where $q'_n$ gathers the expected contributions due to agents' extraction from other sources:

$$q'_n = \sum_{m=1}^{m_{\max}} P_{\mathbf{A}}(m \mid n) \cdot \left[(m-1)\overline{q}_m\right]. \tag{S40}$$

We note that by expressing the time evolution of $\overrightarrow{q}_n$ in Eq. S39, we have implicitly assumed that Eq. S25 describes the time evolution of each $q_{m,n}(t)$ throughout an entire cycle regardless of the values attained; that is, we have neglected the feasibility

cases attempted here.

[5] Alternatively, we could first obtain an expression for $q_{m,n}(t)$ (as in Eq. S43) by integrating Eq. S25 through a depletion-remediation cycle while imposing the boundary conditions that guarantee continuity ($q_{m,n}(T_{\mathrm{V},n}) = \overrightarrow{q}_{\mathrm{D},n}$) and periodicity ($q_{m,n}(0) = q_{m,n}(T_{\mathrm{V},n} + T_{\mathrm{D},n}) = \overrightarrow{q}_{\mathrm{R},n}$). Averaging this expression over a cycle (Eq. S28) reproduces Eq. S37.

condition $q_{m,n}(t) \geq 0$. Integrating Eq. S39 while $\chi = 0$, as $\overrightarrow{q}_n$ increases from $\overrightarrow{q}_{\mathrm{R},n}$ to $\overrightarrow{q}_{\mathrm{D},n}$, yields a predicted viable-state time of

$$T_{\mathrm{V},n} = \frac{1}{k\gamma} \log\left(\frac{n\left[\alpha - \gamma q'_n\right] - \gamma \overrightarrow{q}_{\mathrm{R},n}}{n\left[\alpha - \gamma q'_n\right] - \gamma \overrightarrow{q}_{\mathrm{D},n}}\right). \tag{S41}$$

Similarly integrating Eq. S39 while $\chi = 1$, as $\overrightarrow{q}_n$ decreases from $\overrightarrow{q}_{\mathrm{D},n}$ to $\overrightarrow{q}_{\mathrm{R},n}$ yields a predicted depleted-state time of

$$T_{\mathrm{D},n} = \frac{1}{k\gamma} \log\left(\frac{n\left[\alpha - \beta - \gamma q'_n\right] - \gamma \overrightarrow{q}_{\mathrm{D},n}}{n\left[\alpha - \beta - \gamma q'_n\right] - \gamma \overrightarrow{q}_{\mathrm{R},n}}\right). \tag{S42}$$

Having expressed each $T_{\mathrm{V},n}$ and $T_{\mathrm{D},n}$, and thus each $\overline{\chi}_n$, in terms of the values $\overline{q}_{m,n}$, Eq. S37 now gives a condition for each $\overline{q}_{m,n}$ in terms of $\overline{\mathbf{q}}$ based on a given set of degree distributions. The solutions of this nonlinear system, under the constraint $\overline{q}_{m,n} \geq 0$, can be approximated numerically using a least-squares solver.

The time evolution of $q_{m,n}(t)$ underlying Eq. S41 and S42 through a depletion-remediation cycle beginning at the moment of remediation ($\overrightarrow{q}_n(0) = \overrightarrow{q}_{\mathrm{R},n}$) is given by

$$q_{m,n}(t) = \begin{cases} \left[\frac{\alpha}{\gamma} - (m-1)\overline{q}_{m,n}\right] - \left[\frac{\alpha}{\gamma} - q'_n - \frac{\overrightarrow{q}_{\mathrm{R},n}}{n}\right] e^{-k\gamma t}, & 0 \leq t \leq T_{\mathrm{V},n} \\ \left[\frac{\alpha-\beta}{\gamma} - (m-1)\overline{q}_{m,n}\right] - \left[\frac{\alpha-\beta}{\gamma} - q'_n - \frac{\overrightarrow{q}_{\mathrm{D},n}}{n}\right] e^{-k\gamma\left(t - T_{\mathrm{V},n}\right)}, & T_{\mathrm{V},n} \leq t \leq T_{\mathrm{V},n} + T_{\mathrm{D},n} \end{cases} \tag{S43}$$

Even when the *time-averaged* values $\overline{q}_{m,n}$ satisfy the constraint $\overline{q}_{m,n} \geq 0$, the corresponding curves $q_{m,n}(t)$ may thus dip into negative values, particularly for higher values of agent degree $m$ as extraction pressure approaches the remediation threshold. The estimates produced using this approach thus violate the feasibility condition. Nonetheless, we present these predictions below to help distinguish those aspects of *free adapation* extraction patterns that can be attributed to the form of Eq. S25 alone from those which are consequences of the feasibility condition.

## S3.3 Method 2: Numerical integration with feasibility condition imposed

To analytically integrate Eq. S25 while imposing the feasibility condition $q_{m,n} \geq 0$ presents a difficulty, since the extraction levels $q_{m,n}(t)$ corresponding to different agent degrees $m$ may each reach zero at different times, in turn affecting the times at which $\overrightarrow{q}_n(t)$ (Eq. S29) reaches the thresholds that trigger changes in the source's state $\chi$. To sidestep this complication, we now perform this integration numerically by advancing each $q_{m,n}(t)$ in time for each value of the source degree $n$, using Eq. S25 when applicable and imposing $q_{m,n} \geq 0$ where relevant. Beginning with an initial set of estimates of time-averaged extraction levels $\overline{\mathbf{q}}_0$, we plug this estimate into Eq. S25 to advance each $q_{m,n}(t;\overline{\mathbf{q}}_0)$ in time numerically; here, we use a forward Euler method starting with $q_{m,n}(0) = \left(\overline{q}_{m,n}\right)_0$ as an initial condition, and simulate through several several depletion-remediation cycles of $\overrightarrow{q}_n$ before recording $q_{m,n}(t;\overline{\mathbf{q}})$ and the values $(T_{\mathrm{V},n})_1$ and $(T_{\mathrm{D}},n)_1$ from the final cycle. This $q_{m,n}(t;\overline{\mathbf{q}})$ provides an updated estimate for each $\overline{q}_{m,n}$:

$$\left(\overline{q}_{m,n}\right)_{i+1} = \frac{1}{\left(\left[T_{\mathrm{V},n}\right)_{i+1} + \left(T_{\mathrm{V},n}\right)_{i+1}\right]} \int_0^{\left(T_{\mathrm{V},n}\right)_{i+1} + \left(T_{\mathrm{V},n}\right)_{i+1}} q_{m,n}\left(t;\overline{\mathbf{q}}\right)\, \mathrm{d}t. \tag{S44}$$

These new estimates are then recycled as inputs to Eq. S44 and the process is repeated iteratively until the differences between subsequent iterations $\left(\overline{q}_{m,n}\right)_i$ and $\left(\overline{q}_{m,n}\right)_{i+1}$ are sufficiently small. Results presented below were obtained after $i = 40$ iterations, following an initial guess $\overline{\mathbf{q}}_0$ provided by the outputs of Method 1. Selected curves $q_{m,n}(t)$ and $\overrightarrow{q}_n(t)$ for various source/agent degrees and network types computed using these results are presented in the article (Fig. 3a-c).

## S3.4 Results

### *S3.4.1 Uniform-capacity sources*

We apply the above methods to the case of *uniform capacity* sources considered within the article, where all sources are assumed to share the same depletion and remedation thresholds regardless of their degrees: $\overrightarrow{q}_{\mathrm{D},n} \equiv \overrightarrow{q}_{\mathrm{D}} = 1$ and $\overrightarrow{q}_{\mathrm{R},n} \equiv \overrightarrow{q}_{\mathrm{R}} = .001$. Parameter values are set to $\alpha = 1$, $\beta = 1$, and $\gamma = 0.2$. Using degree distributions extracted from the 9 network ensembles described in Section S1.1, and applying the approximations $P_{\mathbf{S}}(n \mid m) = P_{\mathbf{S}}(n)\frac{n}{\langle n \rangle}$ and $P_{\mathbf{A}}(m \mid n) = P_{\mathbf{A}}(m)\frac{m}{\langle m \rangle}$, we compute estimates as detailed above using Python 3.7.3 with SciPy 1.6.2[6]. We present estimates for $\overline{q}_{m,n}$ provided by the two methods detailed above alongside ensemble mean results from simulations on networks (Fig. S11), along with the corresponding time-averaged quantities: the expected extraction pressure (Fig. S12), quality (Fig. S13), and depleted-state times (Fig. S14) of sources, as well as the expected extraction intensity exerted (Fig. S15) and payoffs extracted (Fig. S16) by agents. These results are discussed in the article. Deviations of the HMF-based predictions from the ensemble means observed from simulation results are discussed below in Section S4.

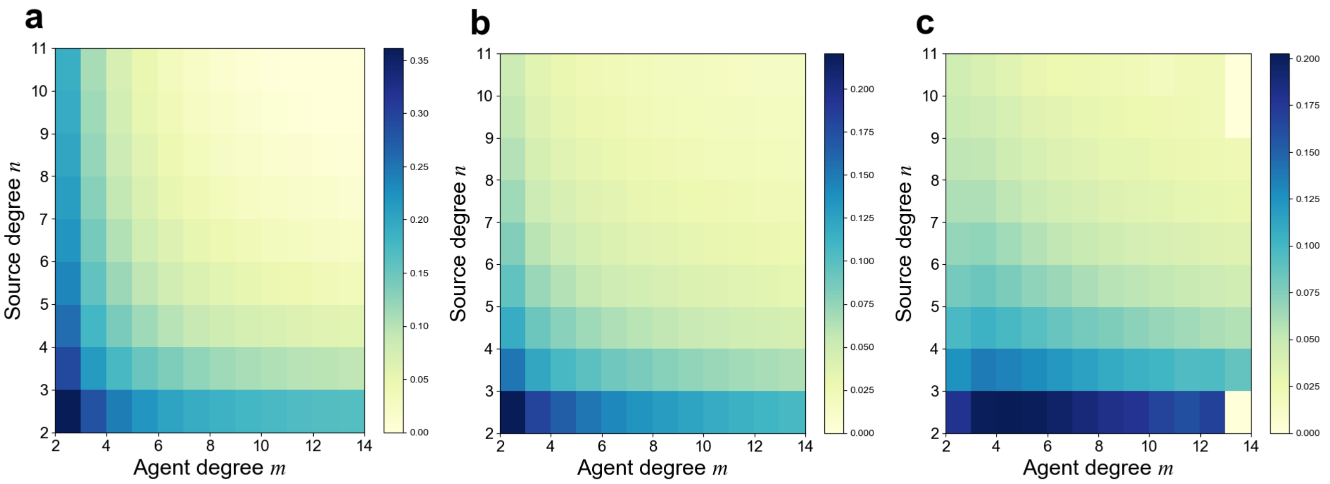


**Figure S11. Time-averaged extraction effort** by degree-$m$ agents on degree-$n$ sources, $\overline{q}_{m,n}$, under *free adaptation* dynamics on **L-l** networks with *uniform capacity* sources: **(a) HMF method 1**, **(b) HMF method 2**, and **(c) Ensemble mean values** from simulations.

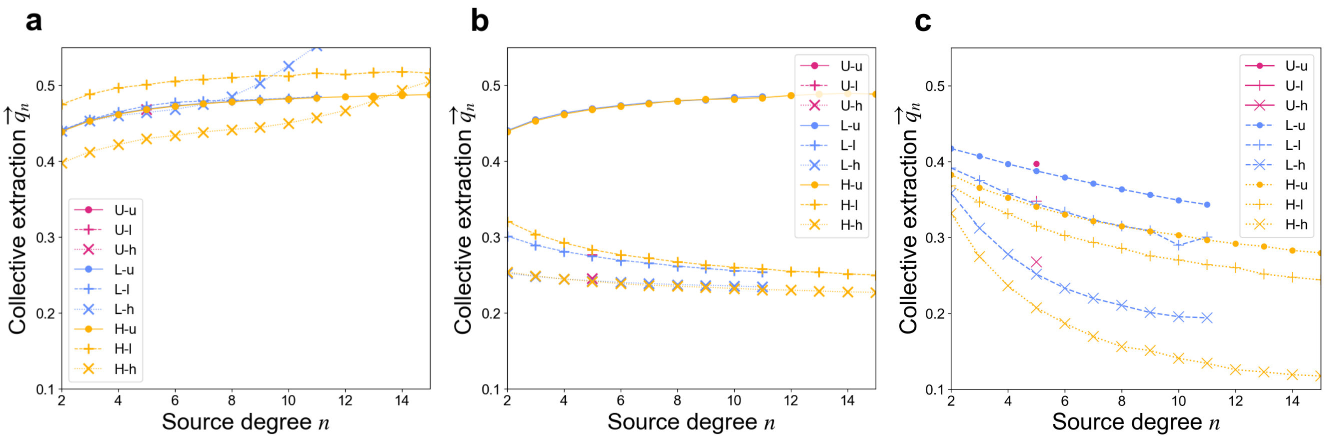


**Figure S12. Time-averaged extraction pressure** $\overrightarrow{q}_n$ on degree-$n$ sources under *free adaptation* dynamics on **L-l** networks with *uniform capacity* sources: **(a) HMF method 1**, **(b) HMF method 2**, and **(c) Ensemble mean values** from simulations.

### *S3.4.2 Degree-proportional capacity sources*

Following previous work on networked CPR extraction games[1], we now use the HMF approach to consider an opposite extreme case of sources with *degree-proportional capacity*, where the values of depletion and remediation thresholds are proportional to the source's degree, so that $\overrightarrow{q}_{\mathrm{D},n} = \overrightarrow{q}_{\mathrm{D}} \frac{n}{\langle n \rangle}$ and $\overrightarrow{q}_{\mathrm{R},n} = \overrightarrow{q}_{\mathrm{D}} \frac{n}{\langle n \rangle}$, with $\overrightarrow{q}_{\mathrm{D}} = 1$ and $\overrightarrow{q}_{\mathrm{R}} = .001$. Under this scenario, the expected viable-state times (Eq. S41) and depleted-state times (Eq. S42) computed using Method 1 are stripped of any explicit dependence on source degree. For example, Eq. S41 becomes

$$T_{\mathrm{V},n} = \frac{1}{k\gamma} \log \left( \frac{\langle n \rangle \left[\alpha - \gamma q_n'\right] - \gamma \overrightarrow{q}_{\mathrm{R}}}{\langle n \rangle \left[\alpha - \gamma q_n'\right] - \gamma \overrightarrow{q}_{\mathrm{D}}} \right). \tag{S45}$$

Furthermore, any potential implicit dependence on source degree (via $q_n'$, Eq. S40) is removed insofar as the approximation $P_{\mathbf{A}}(m \mid n) = P_{\mathbf{A}}(m) \frac{m}{\langle m \rangle}$ can be applied, that is, insofar as there is no degree-based preferential attachment inherent in the network. Sources of all degrees are thus predicted to share a common expected viable-state time $T_{\mathrm{V}}$, depleted-state time $T_{\mathrm{D}}$, expected state $\overline{\chi}$, and expected quality value $\overline{b}$. Eq. S37 then predicts expected extraction levels given by

$$\overline{q}_{m,n} = \frac{1}{m} \left( \frac{\alpha - \beta \overline{\chi}}{\gamma} \right). \tag{S46}$$

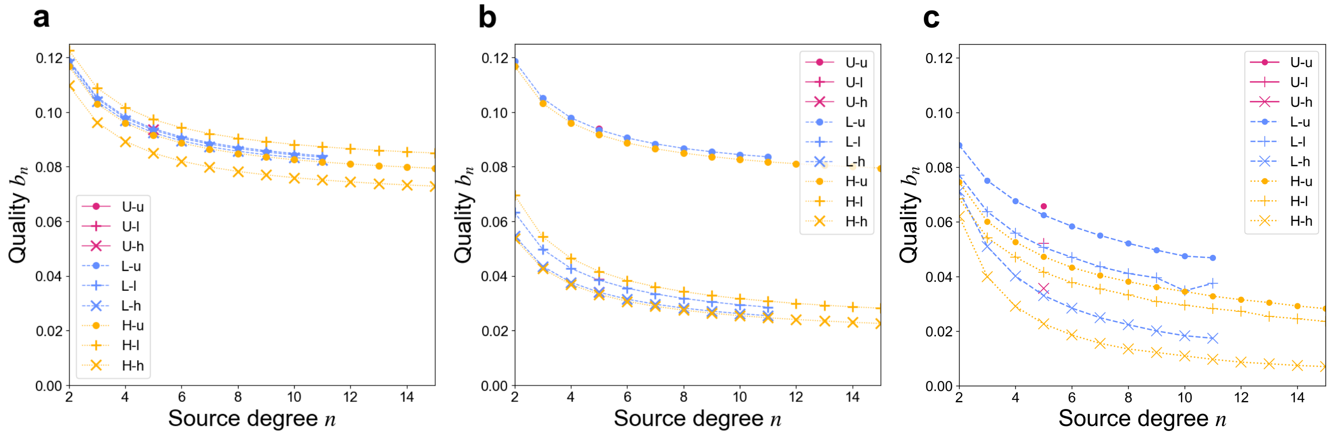


**Figure S13.** **Time-averaged quality** $b_n$ of degree-$n$ sources under *free adaptation* dynamics on **L-l** networks with *uniform capacity* sources: **(a) HMF method 1**, **(b) HMF method 2**, and **(c) Ensemble mean values** from simulations.

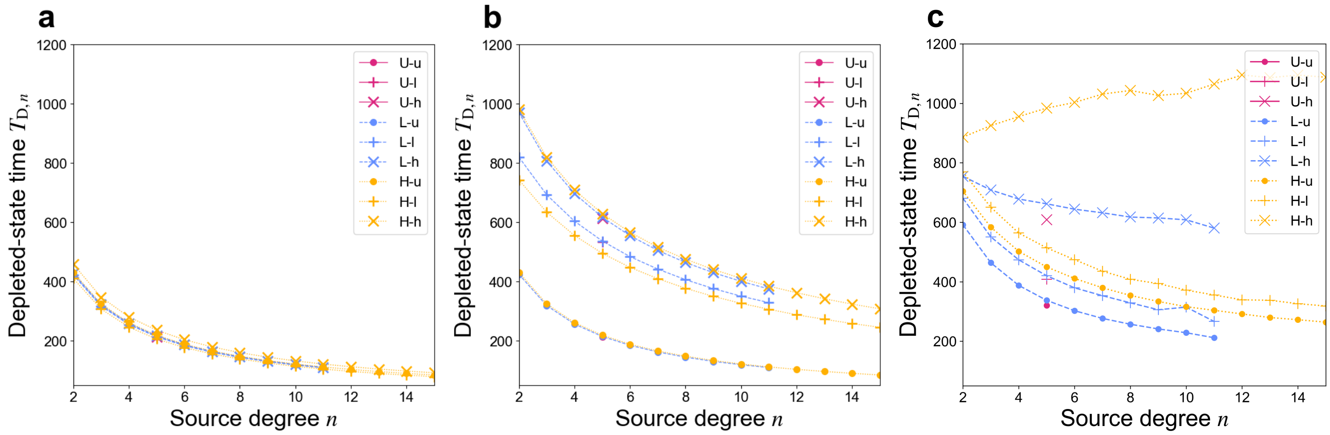


**Figure S14.** **Depleted-state time** $T_{D,n}$ of degree-$n$ sources under *free adaptation* dynamics on **L-l** networks with *uniform capacity* sources: **(a) HMF method 1**, **(b) HMF method 2**, and **(c) Ensemble mean values** from simulations.

An agent's expected extraction intensity $\overleftarrow{q}_m = (\alpha - \beta\overline{\chi})/\gamma$ is thus independent of its degree $m$; in a coarse-grained, time-averaged sense, at least, Method 1 thus predicts that *free adaptation* dynamics here satisfy a *fixed-allocation* assumption: $\overline{q}_{m,n} = \overleftarrow{q}_m/m$ (see also Eq. S13). Although all degree classes of agents share the same expected total extraction intensity $\overrightarrow{q}_m$, the expected payoffs (Eq. S35) they receive from these efforts increase linearly with degree. This is because higher-degree agents expend a greater fraction of this effort upon sources when they are viable, and so waste relatively less effort extracting from depleted sources; specifically, the time-averaged viable- and depleted-state extraction levels are of the form $\overline{q}_{V,n} = \overline{q}_{m,n} + q_0/(k\gamma T_V)$ and $q_{D,n} = \overline{q}_{m,n} - q_0/(k\gamma T_D)$ where $q_0 = [\beta k T_V \overline{\chi} - (\overrightarrow{q}_D - \overrightarrow{q}_R)]$. When applying Method 2, which enforces the feasibility condition $q_{m,n} \geq 0$, several of these patterns persist: expected quality remains independent of source degree (Fig. S17f). However, the faster reduction of extraction levels to zero by higher-degree agents again results in increasing individual extraction with degree (Fig. S17g). Nonetheless, payoffs still appears to increase linearly with agent degree (Fig. S17h), and so higher-degree agents are still expected to extract greater payoffs per link.

Based on these HMF model predictions, we predict that when source capacity is proportional to degree, source degree heterogeneity plays a greatly reduced role compared to the case of *uniform capacity* sources studied in the article; all sources will share approximately similar quality values. Agent degree heterogeneity may lead to slightly decreased overall extraction, reduced resource quality, and reduced collective wealth; the differences between agent extraction and payoff curves for different network types are qualitatively similar to those from the *uniform capacity* source.

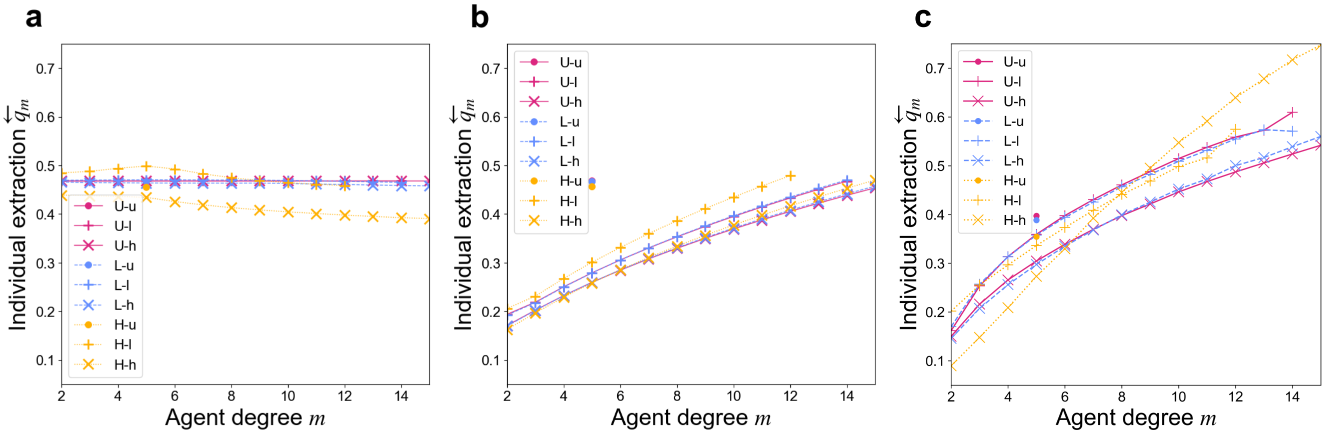


**Figure S15. Time-averaged extraction intensity** $\overleftarrow{q}_m$ of degree-$m$ agents under *free adaptation* dynamics on **L-l** networks with *uniform capacity* sources: **(a) HMF method 1**, **(b) HMF method 2**, and **(c) Ensemble mean values** from simulations.

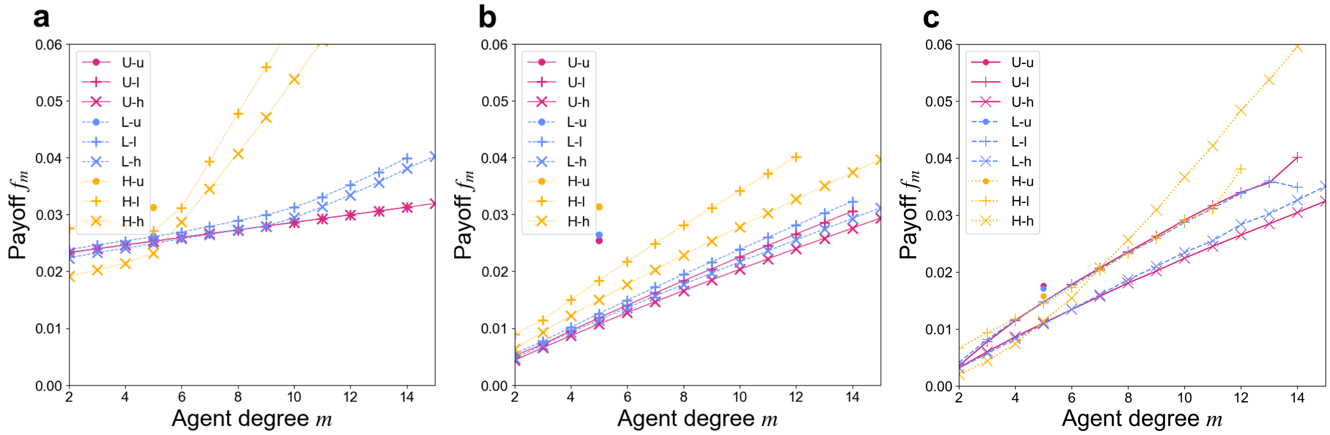


**Figure S16. Time-averaged payoffs** $f_m$ to degree-$m$ agents under *free adaptation* dynamics on **L-l** networks with *uniform capacity* sources: **(a) HMF method 1**, **(b) HMF method 2**, and **(c) Ensemble mean values** from simulations.

## S4 Role of source degree heterogeneity

### S4.1 Limitations of HMF model in explaining observed role of source degree heterogeneity

The heterogeneous mean-field approach (Section S3) succeeds in predicting many of the qualitative trends observed in simulations of free adaptation dynamics, particularly with regards to the role of agent degree heterogeneity. However, it fails to capture the relative shifts in sources' collective extraction and quality levels between networks with different types of source degree heterogeneity as observed in simulations. As such, the HMF approach alone does not help in explaining these observations. The differences between HMF predictions and simulation results with regards to collective extraction and quality (Figs. S12 and S13) trace back specifically to differences between sources' depleted-state times (Fig. S14) in predictions and simulations. The extent of this underestimation is greater at higher-degree sources within networks with high agent degree heterogeneity (Fig. S14). In the extreme case of networks with the highest source and agent degree heterogeneity (**H-h**), simulations show an *increasing* trend of depleted-state time with source degree which the HMF approach completely fails to capture. Here, we investigate this discrepancy.

The evolution of collective extraction from a source $s$ depends on its affiliated agents' fluctuating extraction levels $q(a,s')$ from other sources $s' \neq s$ (Equation S25):

$$\frac{\mathrm{d}}{\mathrm{d}t}\overrightarrow{q}(s) = k\left[n(s)\cdot[\alpha-\beta\chi(s)] - \gamma\sum_{a\in\mathbf{A}_s}\left(H\left(q(a,s)\right)\cdot\sum_{s'\in\mathbf{S}_a\setminus\{s\}} q(a,s')\right) - \gamma\overrightarrow{q}(s)\right] \tag{S47}$$

$$= k\left[n(s)\cdot[\alpha-\beta\chi(s)] - \gamma n(s)q'(s) - \gamma\overrightarrow{q}(s)\right], \tag{S48}$$

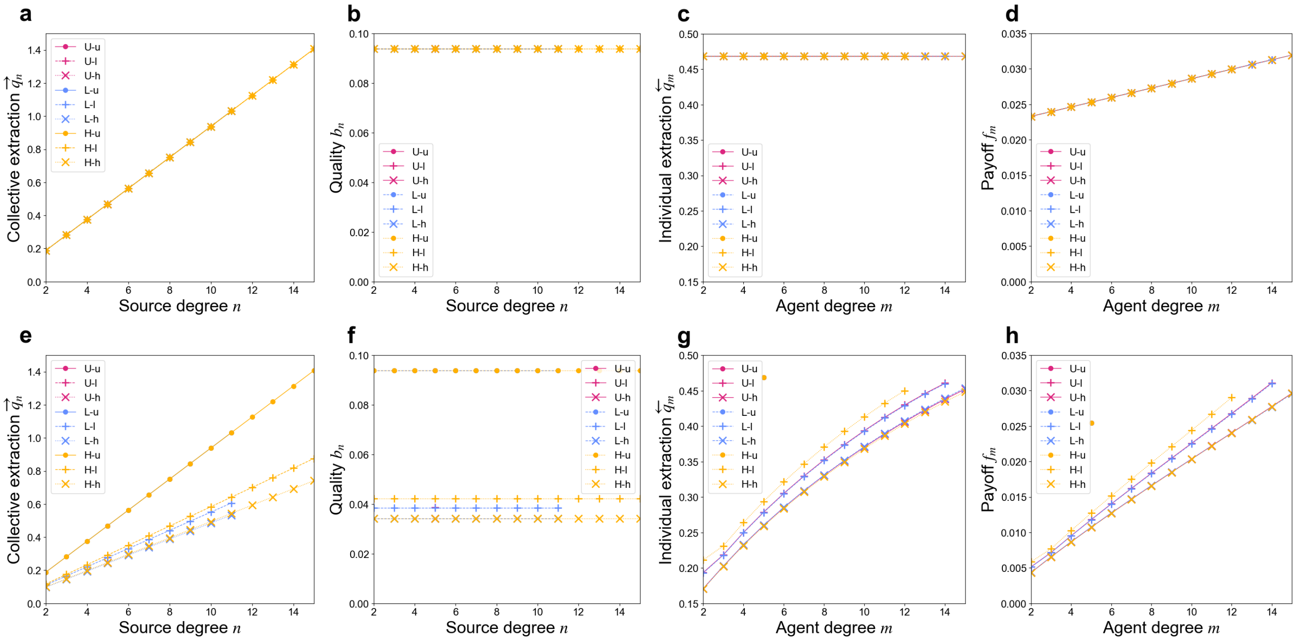


**Figure S17.** ***Free adaptation*** **dynamics of games with** ***degree-proportional capacity*** **sources**. **HMF method 1**: **(a)** Collective extraction $\overrightarrow{q}_n$, **(b)** Source quality $b_n$, **(c)** Individual extraction $\overleftarrow{q}_m$, and **(d)** Payoffs $f_m$; and **HMF method 2**: **(e)** Collective extraction $\overrightarrow{q}_n$, **(f)** Source quality $b_n$, **(g)** Individual extraction $\overleftarrow{q}_m$, and **(h)** Payoffs $f_m$.

where $q'(s) = \left[\sum_{a\in\mathbf{A}_s} H\left(q(a,s)\right)\cdot\left(\sum_{s'\in\mathbf{S}_a\setminus\{s\}} q(a,s')\right)\right]/n(s)$ and $H(x) = 1$ if $x > 0$ and $H(x) = 0$ otherwise. In the HMF approach, we approximated these ambient extraction levels for sources of each degree $n$ by using their time-averaged values ($q'_n \to \langle q'_n\rangle$). We then used Equation S47 (recast as Equation S39) to simulate a source's "representative" time evolution through a cycle of depletion and remediation within a given network type. In this way, we used a set of estimates for ambient extraction values $\langle q'_n\rangle$ to compute the depleted-state time $T_{\mathrm{D},n}$.

If indeed we consider a source's depleted-state time $T_{\mathrm{D},n}$ as a function of these ambient extraction values ($T_{\mathrm{D},n} = T_{\mathrm{D},n}\left(q'_n\right)$), we find that this function is highly convex with respect to $q'_n$. For example, the closed-form expression for $T_{\mathrm{D},n}$ obtained using HMF Method 1 (Equation S42) is a convex function of $q'_n$, increasing sharply as $q'_n$ decrease towards zero, but then decreasing gradually as $q'_n$ approaches higher values. This predicted convex trend is confirmed in simulation results when each source's depleted-state times $T_{\mathrm{D}}(s)$ are plotted against the ambient extraction values averaged over the corresponding depleted-state phase (Fig. S20). We heuristically argue that insofar as $T_{\mathrm{D},n}$ can be considered as a function of these ambient extraction values, then it is a convex function; applying Jensen's inequality[7],

$$T_{\mathrm{D},n}\left(\langle q'_n\rangle\right) < \langle T_{\mathrm{D},n}\left(q'_n\right)\rangle, \tag{S49}$$

leads us to expect that the HMF approach will underestimate the mean values of the depleted-state times $T_{\mathrm{D},n}$. That is to say, the average values of depleted-state times $\langle T_{\mathrm{D},n}\left(q'_n\right)\rangle$ observed in simulations will exceed the values predicted by replacing the actual fluctuating values $q'_n$ with their time-averaged values $\langle q'_n\rangle$, as we have done in our HMF estimates. In this sense, the underestimation of $T_{\mathrm{D},n}$, and the corresponding overestimation of source quality $b_n$, is to be expected from the convex dependence of depleted-state times on ambient extraction.

These extent of these discrepencies in depleted-state times affect sources of different degrees in the different ways, and differ for networks with different types of degree heterogeneity. Since these effects depend on couplings between sources of different degrees, we now clarify some of these couplings through games played out on simpler *star networks*, consisting of a single higher-degree focal source situated among agents linked to lower-degree peripheral sources.

## S4.2 Illustration using bipartite star networks

We now consider networks consisting of a *focal* source of degree $n_{\mathrm{f}}$ whose $n_{\mathrm{f}}$ affiliated agents, each with degree $m = 3$, are also linked to degree-2 *peripheral* sources (Fig. S18). We play out the CPR extraction games (with $u = 1$ and $k = .01$, computing averages over the final $9\times10^3$ iterations of simulations lasting $10^4$ iterations) on star networks with a range of values of the focal source degree $n_{\mathrm{f}}$, over a number of trials (60) with *randomized* initial conditions. In order to illustrate an exaggerated case where ambient extraction values often attain more extreme values, we also consider *synchronized* initial

conditions where all peripheral sources begin with the same collective extraction. For comparison, we also apply the HMF model (Method 2) to degree distributions representing these star networks ($P_{\mathbf{S}}(n) = \left(\frac{n_f}{n_f+1}\right)\delta_{2,n} + \left(\frac{1}{n_f}\right)\delta_{n_f,n}$, $P_{\mathbf{A}}(m) = \delta_{3,m}$, $P_{\mathbf{S}}(n \mid m=3) = \frac{2}{3}\delta_{2,n} + \frac{1}{3}\delta_{n_f,n}$).

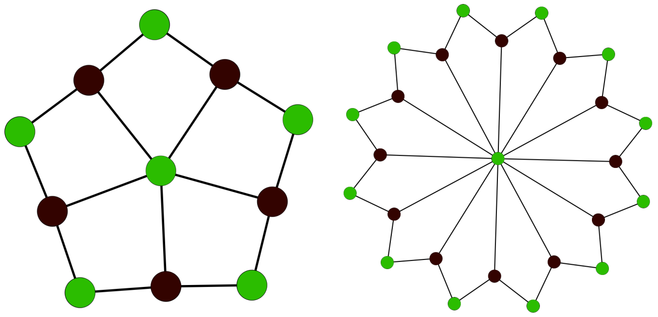

**Figure S18.** Bipartite star networks of sources (green) and agents (black) with focal source degree $n_f = 5$ **(left)** and focal source degree $n_f = 12$ **(right)**.

The higher the degree of the focal source that is situated among more numerous low-degree sources, the lower agents' overall extraction (Fig. S19a and e). Due to the construction of these star networks, as focal source degree is increased, the overall number of sources per agent $\frac{n_f+1}{n_f}$ decreases slightly. However, the network's total extraction—and total extraction *per source*, and the extraction from each peripheral source—also decreases. Just as we observed in more complex networks, source quality declines as source degree increases (Fig. S19b and f). Also as in more complex networks, overall time-averaged collective extraction from the focal source also decreases as degree increases, and each agent accounts for a smaller relative share of this extraction ($\propto \frac{1}{n_f}$). This reduction in agents' focal-source extraction leads them to increase their extraction more rapidly at viable peripheral sources, decreasing their viable-state times, and reduce their extraction more slowly at peripheral sources, increasing their depleted-state times (Fig. S19c and g). While the HMF model correctly captures most of the qualitative features of these trends, it tends to underestimate depleted-state times and overestimate source quality.

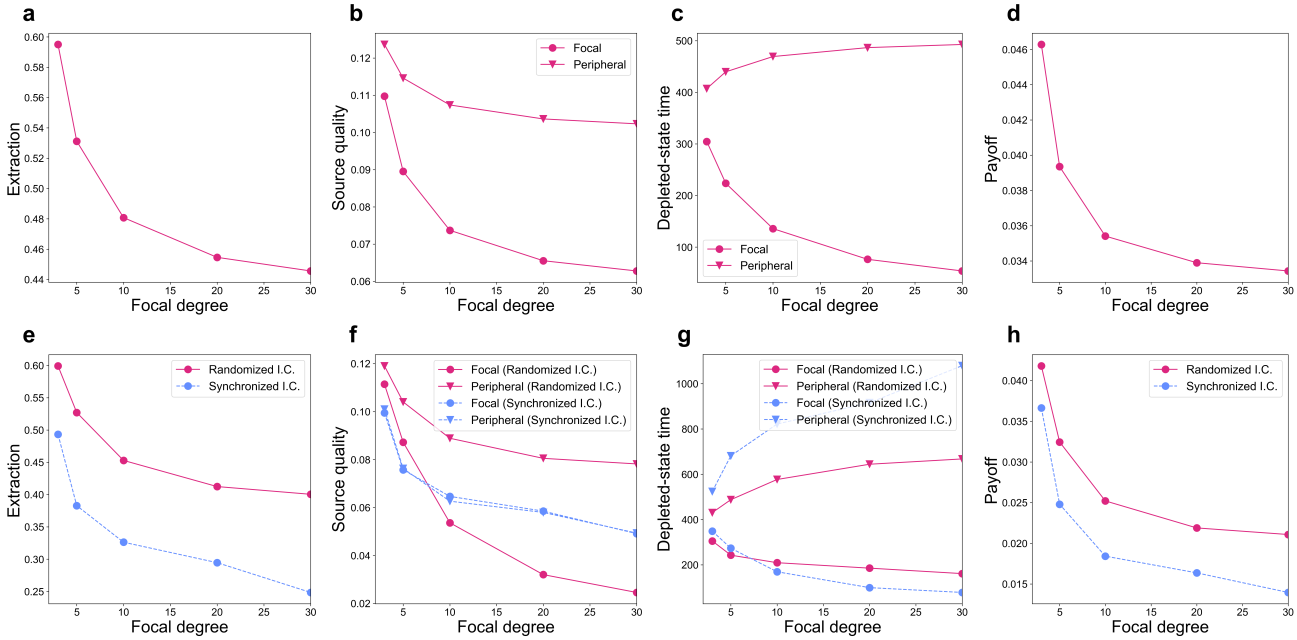


**Figure S19. Star network results** from 60 simulations on networks of each focal degree. **Heterogeneous mean-field estimates**: **(a)** Individual agent per agent, **(b)** Source quality, **(c)** Depleted-state times, and **(d)** Payoff per agent; **Ensemble mean simulation results**: **(e)** Individual agent per agent, **(f)** Source quality, **(g)** Depleted-state times, and **(h)** Payoff per agent.

### S4.3 Illustration within a complex network context

As dicussed above, the deviations of simulation results from the corresponding HMF predictions can largely be understood in terms of the convex dependence of depleted-state times on ambient extraction values. To illustrate the variability among depleted state times for sources of different degrees within different network types, we plot depleted-state times against the time-averaged values of ambient extraction (see Eq. S47) in Fig. S20. In networks with uniform agent degree ("**u**"), ambient extraction levels vary within relatively narrow bands at magnitudes proportional to their degrees (Fig. S20a and b).

The presence of agent degree heterogeneity allows ambient extraction levels to dip into lower ranges. As illustrated in the article (Fig. 2a), in networks with agent degree heterogeneity, higher-degree agents tend to reduce their extraction levels from depleted sources to zero. For the remainder of that source's duration in the depleted state, the source's remaining collective extraction is then carried by its more lower-degree users, who reduce extraction more slowly due to their lower overall extraction levels. The presence of higher-degree agents means that the effective number of agents contributing to ambient extraction is reduced throughout most of a source's depleted-state phase. The fact that those agents that continue to extract from the source are of low degree means that the contributions of each agent to ambient extraction will tend to remain small. This allows ambient extraction values to attain lower values, so that depleted-state times are extended (Fig. S20c and d).

In networks with the highest agent degree heterogeneity, this becomes even more extreme (Fig. S20e and f). As previously observed (Fig. S14), the highest-degree sources in **H-h** networks actually have longer depleted-state times than do lower-degree sources, contrary to the trend observed for all other network types. Very high-degree sources can become depleted so quickly that they often overshoot the depletion threshold before agents begin to react to depletion, and so often require a significant amount of additional time to reduce extraction through this greater range before triggering remediation. However, Fig. S20f also illustrates that in these networks, the highest-degree sources are indeed restricted to a lower range of ambient extraction values. Within these networks, where low-degree sources and agents are most abundant, a low-degree source is likely to have all low-degree users. Higher-degree sources, however, are basically guaranteed to share some links with the network's highest-degree agents, while sharing other links with low-degree agents. This leads these high-degree sources' ambient extraction to attain lower values despite their many links, further enhancing and widening the gap described by Jensen's inequality (Eq. S49). An abundance of lower-degree sources, at which collective extraction evolves much more slowly than at higher-degree sources, results in ambient extraction often remaining at lower levels throughout the course of one or more depletion-remediation cycles of these higher-degree sources.

## S5 Effects of changes to parameter values

### S5.1 Cost parameter

In this model, the changes in extraction costs that agents experience as they vary their extraction levels are the dominant factor shaping dynamics in the times between regime shifts in resource quality. These changing costs are the reason that network degree heterogeneity plays a role in shaping a system's dynamics and wealth distributions. To estimate how results would be affected by changes in the cost parameter $\gamma$, we apply our heterogeneous mean-field technique to predict free adaptation extraction patterns that result for a smaller value ($\gamma = 0.1$) and a larger value ($\gamma = 0.4$) of the cost parameter than that presented in the article ($\gamma = 0.2$) but with all other parameters settings held fixed. The resulting estimates for the $\gamma = 0.1$ case (Fig. S21) and the $\gamma = 0.4$ case (Fig. S22) suggest that increasing the cost parameter raises the overall quality of sources, as greater costs lead to slower depletion and faster remediation of sources. Overall extraction levels remain in similar ranges (except perhaps for "**h**" networks with the greatest agent degree heterogeneity), and so increased costs lead to greater payoffs. Since the effects of degree heterogeneity here all result from how increasing marginal costs differently affect agents of different degrees, increased values of $\gamma$ also further exaggerate the distinctions between networks with different types of degree heterogeneity (Fig. S23). In each of these cases, the relative differences between different network types are consistent, predicting that the qualitative results reported in the article are not altered by changes in $\gamma$.

### S5.2 Remediation and depletion thresholds

Setting the remediation threshold near zero ($\overleftarrow{q}_{\mathrm{R}} = .001$) as in the article requires agents to reduce extraction almost completely before a source can be remediated. In the article, several features of dynamics are attributed to these near-zero extraction values: for example, (1) higher-degree agents quickly reduce extraction to zero when a source is depleted, and (2) low-degree agents drastically delay source remediation since their individual extraction levels become so low. It seems that shifting the remediation threshold $\overrightarrow{q}_{\mathrm{R}}$ away from zero extraction towards larger values could potentially affect both of these findings.

To investigate, we apply the heterogeneous mean-field technique to estimate how dynamics are altered if both thresholds are shifted upwards by the same amount: by 0.05 extraction units ($\overrightarrow{q}_{\mathrm{R}} = .05$, $\overrightarrow{q}_{\mathrm{D}} = 1.05$) in the first case, and further by 0.1 ($\overrightarrow{q}_{\mathrm{R}} = .1$, $\overrightarrow{q}_{\mathrm{D}} = 1.1$) in a second case. The resulting predicted time evolution of extraction levels shows that high-degree agents still quickly reduce their extraction to zero upon resource depletion, and so still sustain zero extraction for a large fraction of a source's depleted-state phase (Fig. S24a). However, low-degree agents, whose extraction levels are still well above zero

when the source becomes remediated, now sustain elevated extraction levels throughout each depletion-remediation cycle. This boosts individual extraction (Fig. S26b and Fig. S27b) and payoffs (Fig. S26c and Fig. S27c) for lower-degree agents, while leaving higher-degree agents less affected. Although this somewhat alters the dependence of agents' individual extraction and payoffs upon degree, the relative shifts between networks with different types of degree heterogeneity resemble those observed at lower threshold values. This elevation of individual extraction levels for the lowest-degree agents de-emphasizes the aforementioned slowing effect, illustrated by tapered extraction decay curves (Fig. S24b versus Fig. S25b), that delays source remediation and so reduces quality in the presence of high agent degree heterogeneity. This makes the reductions in resource quality associated with higher agent degree heterogeneity less pronounced (Fig. S26b versus Fig. S27b).

### S5.3 Quality parameters

Our choice of parameter values $\beta = \alpha$ represents complete resource depletion. Under *free adaptation* dynamics (Eq. S8), this choice ensures that agents will reduce their extraction levels from a depleted source, regardless of other parameter settings. As a result, collective extraction levels from any depleted source will always eventually reach the remediation threshold. A value of the parameter $\beta < \alpha$ would mean that depleted source offers a reduced, but still positive, benefit. This introduces a qualitative change to the system's behavior since an agent's extraction levels may now stagnate if the marginal benefit of extraction from a depleted source balances the marginal cost, that is, if

$$\overleftarrow{q}(a) = \frac{\alpha - \beta}{\gamma}. \tag{S50}$$

How readily these dynamics stagnate or sustain repeated depletion-remediation cycles would seem to depend largely on the value of sources' remediation thresholds $\overrightarrow{q}_{\mathrm{R}}$. If thresholds are high enough that extraction levels fall below them before reaching the stagnation levels indicated by Eq. S50, then cyclical depletion-remediation dynamics could still occur. While a full treatment of this more general case is beyond the scope of the current analysis, we note the similarity of this problem to one studied previously[1,3] — that of finding the steady states where agents' marginal payoffs balance marginal costs on a network — with the added complication, from the mean-field perspective, of estimating expected source states $\overline{\chi}_n$ based on expected extraction levels $\overrightarrow{q}_n$.

### S5.4 Rate constant

The rate constant $k$ determines the overall rates at which agents change their extraction levels in all update rules (Eq. S8, Eq. S11, and Eq. S16). An increase in this parameter speeds up dynamics by an overall factor of $k^{-1}$, and so reduces each source's viable-state times and depleted-state times (as is visible in Eq. S41 and Eq. S42). However, this does not alter the *relative* amounts of time spent in each state. Similarly, the characteristic "shapes" of sources' depletion-remediation waveforms are not distorted, but merely rescaled by such a change. Since these relative times and waveforms determine the time-averaged quantities of interest, such as source quality (see Eq. S30, where the factor of $k^{-1}$ from $T_{\mathrm{D},n}$ and $T_{\mathrm{V},n}$ vanishes) and average extraction, these quantities will be unaffected by changes in the overall rate constant $k$. However, within a time-discretized scheme such as that used here (in the discrete iterations of our simulations, and in the forward Euler scheme used in the heterogeneous mean-field technique), a higher value of $k$ relative to the time step could lead to extraction levels "overshooting" thresholds by larger amounts (as visible in Fig 3d,e). Whether these are seen as mere numerical artifacts, or as meaningful overshoots that could occur if agents change extraction levels very quickly relative to the rates at which they receive new information about resource quality, is a matter of intepretation.

## S6 Relaxing the cooperator/defector dichotomy in networked PGGs with imitative dynamics

### S6.1 Role of the uniform-allocation assumption

In the current article and in previous work[1], we argued heuristically that the major results of networked *public goods game* (PGG) models depend on what we might call a *uniform-allocation* assumption with respect to agents' contributions to PGGs. In these games, agents are only permitted to either *cooperate* (make a contribution of fixed size) or *defect* (withold all contributions) with respect to *all* of their affiliated public goods simultaneously. In these models, there is no possibility for agents to selectively allocate their contributions, for example by contributing only to those public goods where contribution appears profitable while witholding contributions from other goods where contribution appears less profitable. While a few studies have addressed the possibility that agents' might adaptively vary how they allocate their contributions among goods, we note that in these models, agents still identify as, and perceive one another's strategies in accord with, a cooperator/defector binary. Although an agent's behavior is influenced by imitation of its peers via pairwise fitness comparisons, its *local* behavior (at the source where the pairwise encounter occurs) is not influenced by the peer's *local* contribution behavior; an agent could therefore be influenced

to imitate the "cooperator" identity of a peer who contributes nothing to the PGG that they both share. Abandonment of this assumption, we argue, would largely decouple a network's multiple PGGs from one another, disrupting the mechanism by which cooperation is able to spread in these models.

In these PGG models, unlike the CPR games considered in this article, agents' strategy behavior is assumed to be driven by *imitation*. Cooperation can spread when agents, lacking correct information about how conversion to an alternative strategy would affect their payoffs, attempt to glean this information indirectly by observing the payoffs accumulated by peers who practice the alternative strategy. If an agent observes a peer achieving a higher payoff while practicing the alternative strategy, then the agent — incorrectly attributing this difference to the strategy choice itself, rather than to the peer's involvement in other, more cooperation-dominated games — may adopt the alternative strategy in hopes of achieving a similar outcome for itself. Under this assumption, an agent's imitative adoption of cooperation will simultaneously introduce cooperation into *all* of the agent's affiliated public goods games, improving cooperation levels at these games while also exposing its peers at each public good to the cooperative strategy in their own pairwise comparisons. If the agent's peers then go on to adopt cooperation, the agents' own payoffs will indeed improve, and furthermore the agent will be increasingly insulated from re-exposure to defection by its peers. These myopic agents are attempting only to increase their own immediate individual payoffs; if they had more complete information about their own payoff functions, they would surely notice that cooperation costs more than defection in any given round, and so would *always* defect. However, *misinformation* about the effects of cooperation can lead them to erroneously perceive an incentive to cooperate, and so to imitate cooperative behavior. Due to the *uniform-allocation assumption*, these misguided myopic agents thus inadvertently end up improving the conditions of their affiliated public goods. They thus inadvertently benefit their fellow community members, and also promote cooperation. In this way, the combination of *strategy imitation* based on pairwise fitness comparison with a *uniform-allocation assumption* can make cooperation self-reinforcing within networked PGGs.

If agents do not imitate a higher-fitness peer's overall "cooperator" or "defector" identity, but instead imitate its local contribution behavior only the game where they met, then the uniform-allocation assumption will be completely relaxed, disrupting this positive feedback loop. Introduction of cooperation into one PGG, and even the subsequent adoption of cooperation by other agents that PGG, will no longer also serve to introduce cooperation into each agent's other PGGs. Cooperation (i.e., voluntary contribution to PGGs) will thus no longer spread from site to site through a network. To illustrate this heuristic argument more explicitly, here we simulate an iterative networked PGG game (a multiplayer Prisoner's Dilemma) with and without a *uniform-allocation assumption*.

## S6.2 Model

We consider bipartite networks linking a set of agents $\mathbf{A}$ with a set of nodes $\mathbf{S}$, now interpreted as *public goods* rather than CPR sources. The presence of a link $(a,s)$ means that the agent $a$ has access to the public good $s$, and the flow $q(a,s)$ now represents the magnitude of the *contribution* made by agent $a$ toward a public good $s$ (rather than the "extraction effort" applied by $a$ to $s$ as in the CPR game). In a multiplayer *Prisoner's Dilemma*, the collective contributions to each public good $s$ by its users return a collective payoff, proportional to the original contribution by a *enhancement factor r*; this collective payoff is then shared evenly among the PGG's $n(s)$ affiliated agents. The net payoff received by an agent $a$ following a round characterized by contributions $q(a,s)$ is then given by

$$f(a) = \sum_{s \in \mathbf{S}_a} \left[ \frac{r}{n(s)} \left( \sum_{a' \in \mathbf{A}_s} q(a',s) \right) - q(a,s) \right]. \tag{S51}$$

In accord with typical networked PGG models, we further assume that the values $q(a,s)$ can assume just one of two values: $q(a,s) \in \{0,1\}$. In a typical networked PGG with a *uniform-allocation assumption*, at each iteration, each agent $a$ will …

1. Randomly select a source $s \in \mathbf{S}_a$, from which a peer $a' \in \mathbf{A}_s$ is randomly selected.
2. Observe the peer's fitness and strategy: if $f(a') > f(a)$ and $q(a',s) \neq q(a,s)$, then $a$ updates its strategy **at all affiliated sources** with probability $[f(a') - f(a)]/A$, where $A$ is a normalization factor. That is, it sets $q(a,s') = q(a',s)$ for all $s' \in \mathbf{S}_a$.

In a game where this assumption is relaxed, each agent $a$ can vary its contribution at each public good independently. At each iteration, the agent will …

1. Randomly select a peer $a'_s \in \mathbf{A}_s$ from **each affiliated source** $s \in \mathbf{S}_a$.
2. Observe each peer's fitness and strategy: if $f(a'_s) > f(a)$ and $q(a'_s,s) \neq q(a,s)$, then $a$ updates its strategy with probability $[f(a') - f(a)]/A$, where $A$ is a normalization factor, by setting $q(a,s) = q(a'_s,s)$.

We initialize each simulation so that half of the network's agents "cooperate" (contribute $q(a,s) = 1$) at *all* affiliated sources, while the remaining agents "defect" ($q(a,s) = 0$) at all affiliated sources. For each network, we simulate once *with* a uniform-allocation assumption and once *without*, each time using the same initial condition. Simulations shown use networks from the above ensembles, with $A = 50$.

### S6.3 Simulation results

In the absence of a uniform-allocation assumption, the steady-state fractions of cooperators achieved in networks increases with $r$, and so appears to undergo a transition towards cooperation, albeit not the drastic, sudden transition from universal defection to universal defection observed under a uniform-allocation assumption (Fig. S28). Does this mean that without a *uniform-allocation* assumption, the system behaves in a similar, albeit less-exaggerated way? Closer inspection of simulation results reveals that the results here are qualitatively different; the "transition" observed here merely reflects the slight shifts of cooperation that occur within individual PGGs given the randomized, half-cooperator/half-defector initial conditions used.

Without the uniform-allocation assumption, public goods that are initially dominated by cooperation tend to remain so, and those initially dominated by defection tend to remain so as well, as visible in the clustering of points near the diagonals in Fig. S29a-c. For public goods that begin with a mixture of cooperators and defectors under the half-cooperator/half-defector initial condition used, the enhancement factor $r$ plays a role to tip the balance towards cooperation or defection as it would in any multiplayer Prisoner's Dilemma: for lower values of $r$, cooperation tends to die out in the presence of any amount of defection, while for sufficiently high values of $r$ ($r \geq n(s)$), cooperation (with respect to the shared PGG at hand) can spread to all group members. However, although pairwise fitness comparisons can still incentivize agents to cooperate within individual PGGs, cooperation does not spread from game to game through the network here. This is confirmed by observing that the steady-state fraction of cooperators achieved at each source is determined almost entirely by its initial fraction of cooperators. The transitions to universal cooperation (or defection) observed under the uniform-allocation assumption — where the steady-state fraction of cooperators within a group appears to independent of initial fraction of cooperators (see the shared mean contribution values for all PGGs regardless of their initial mean contributions in Fig. S29a-c) — do not occur here.

In this game, agents' still use a peer's *net fitness*, which includes payoffs accumulated from other PGGs, as a proxy for direct information about the potential benefits of cooperation at the source at hand. This retains a form of "coupling" by which cooperative conditions at one game can induce an agent to cooperate at another. Were this removed, for example if agents were able to observe their peers' payoffs and contributions specifically involved with the source at hand, then the games would become fully decoupled. As we show here, though, granting agents the ability to make contribution decisions separately at each PGG is sufficient to disrupt the spreading reported in previous models.

The extensive literature on networked PGG models reports topology-dependent phase transitions from defection to universal cooperation caused by a site-to-site spread of "cooperation" (voluntary contribution behavior) through the network. The results above explicity confirm that these findings are reliant on a *uniform-allocation assumption*: agents must not selectively vary their contributions among multiple public goods, but must apply the same behavior to *all* games regardless of the different conditions present at each. In situations where individuals do not conform to this assumption, and local contribution behavior is imitated locally, then the conclusions of these models do not apply.

## References


1. Schauf, A. & Oh, P. Myopic reallocation of extraction improves collective outcomes in networked common-pool resource games. *Scientific Reports* **11**, 1–10 (2021). URL http://www.nature.com/articles/s41598-020-79514-5.
2. Ohkubo, J., Tanaka, K. & Horiguchi, T. Generation of complex bipartite graphs by using a preferential rewiring process. *Physical Review E* **72**, 036120 (2005). URL https://link.aps.org/doi/10.1103/PhysRevE.72.036120.
3. İlkılıç, R. Networks of common property resources. *Economic Theory* **47**, 105–134 (2011). URL http://link.springer.com/10.1007/s00199-010-0520-7.
4. Börgers, T. & Sarin, R. Learning Through Reinforcement and Replicator Dynamics. *Journal of Economic Theory* **77**, 1–14 (1997). URL https://linkinghub.elsevier.com/retrieve/pii/S002205319792319X.
5. Lahkar, R. & Seymour, R. M. The dynamics of generalized reinforcement learning. *Journal of Economic Theory* **151**, 584–595 (2014). URL https://linkinghub.elsevier.com/retrieve/pii/S0022053114000039.
6. SciPy 1.0 Contributors *et al.* SciPy 1.0: fundamental algorithms for scientific computing in Python. *Nature Methods* **17**, 261–272 (2020). URL http://www.nature.com/articles/s41592-019-0686-2.
7. Mukhopadhyay, N. *Probability and statistical inference*. No. 162 in Statistics, Textbooks and Monographs (Marcel Dekker, New York, 2000).

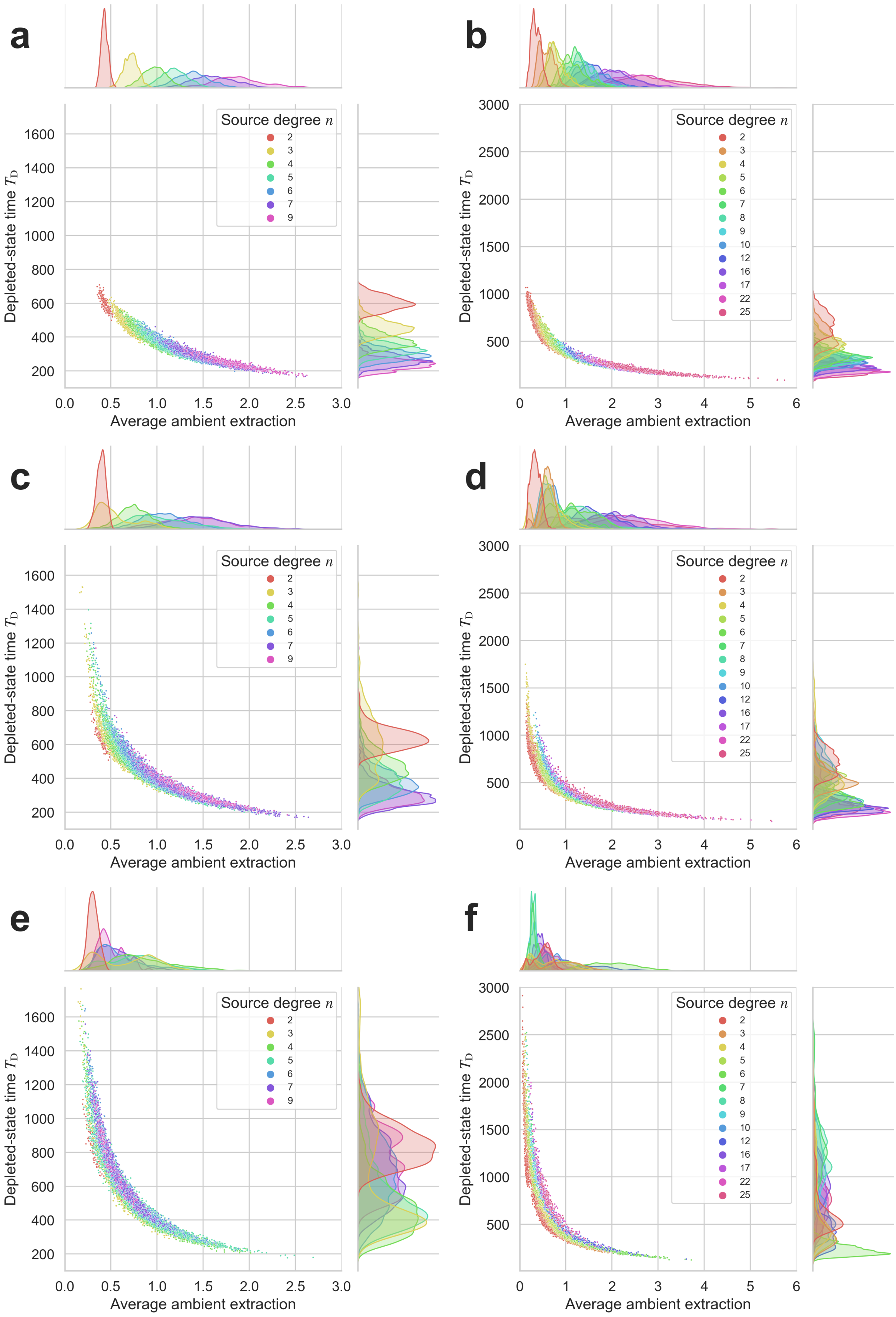


**Figure S20.** **Variability among sources' depleted-state times by degree**. Depleted state times versus ambient extraction values averaged over the corresponding depleted-state duration from simulations on **(a)** an **L-u** network, **(b)** an **H-u** network, **(c)** an **L-l** network, **(d)** an **H-l** network, **(e)** an **L-h** network, and **(f)** an **H-h** network.

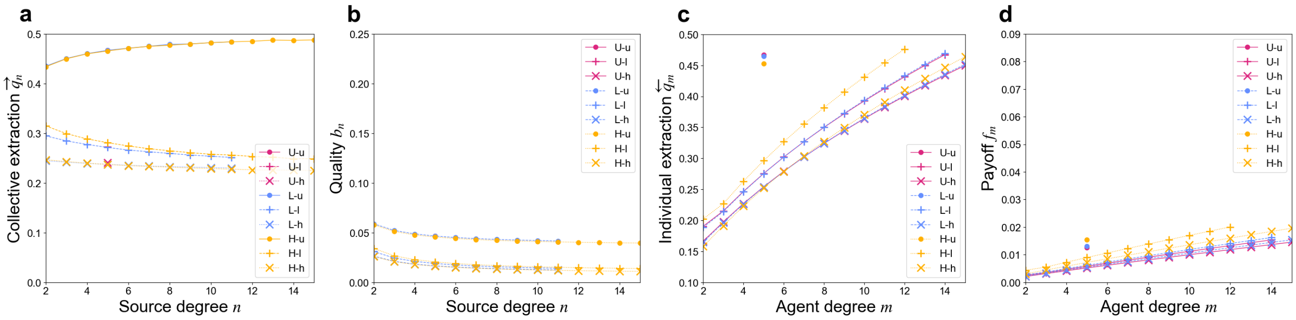


**Figure S21. Decreasing the cost parameter:** $\gamma = 0.1$**.** Time-averaged quantities from *free adaptation* dynamics estimated using HMF method 2: **(a)** Collective extraction $\overrightarrow{q}_n$, **(b)** Source quality $b_n$, **(c)** Individual extraction $\overleftarrow{q}_m$, and **(d)** Payoffs $f_m$. Other parameter settings remain as before: $\alpha = \beta = 1$, $\overrightarrow{q}_{\mathrm{R},n} = .001$, and $\overrightarrow{q}_{\mathrm{D},n} = 1$.

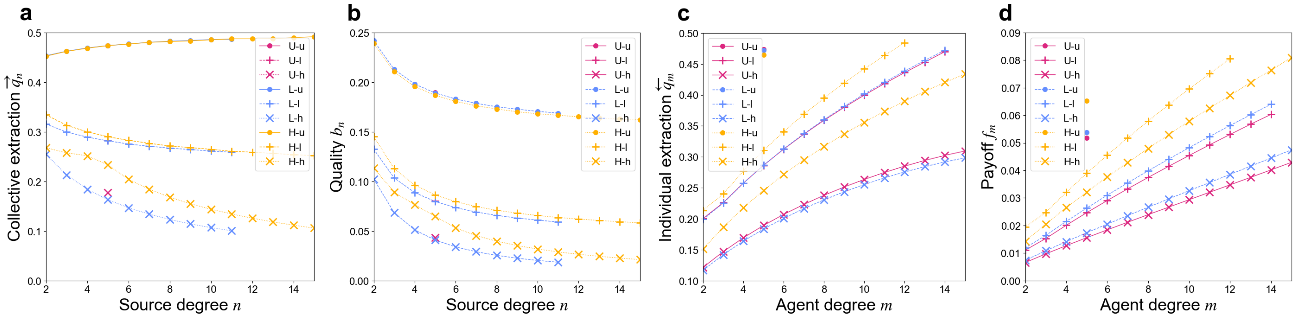


**Figure S22. Increasing the cost parameter:** $\gamma = 0.4$**.** Time-averaged quantities from *free adaptation* dynamics estimated using HMF method 2: **(a)** Collective extraction $\overrightarrow{q}_n$, **(b)** Source quality $b_n$, **(c)** Individual extraction $\overleftarrow{q}_m$, and **(d)** Payoffs $f_m$. Other parameter settings remain as before: $\alpha = \beta = 1$, $\overrightarrow{q}_{\mathrm{R},n} = .001$, and $\overrightarrow{q}_{\mathrm{D},n} = 1$.

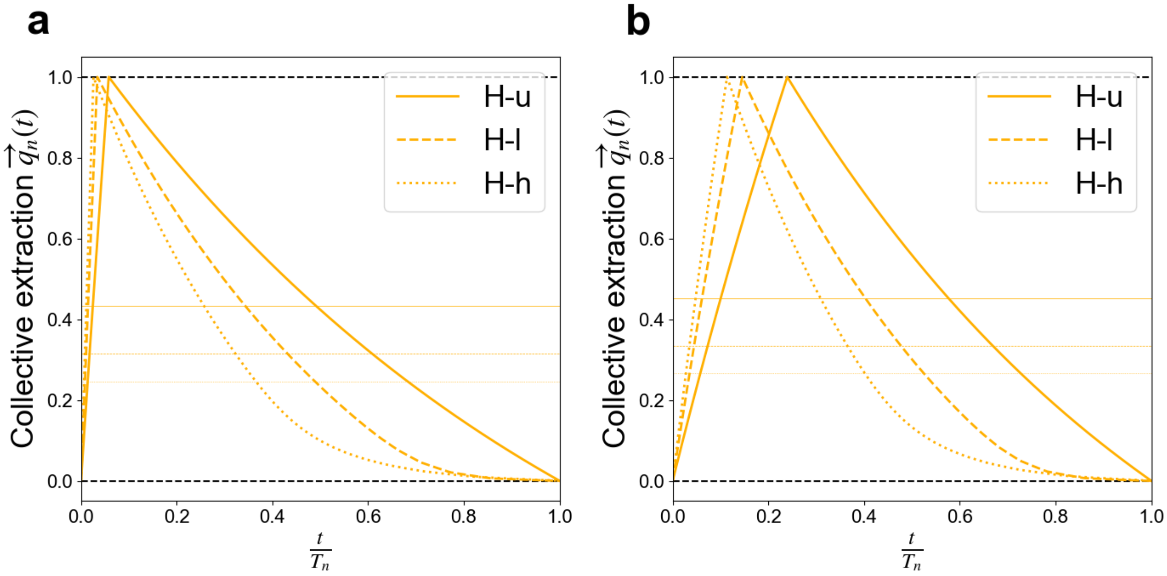


**Figure S23.** HMF model predicted time evolution of collective extraction for networks with different types of agent degree heterogeneity and high source degree hetereogeneity (**H**) for **(a)** $\gamma = 0.1$, and **(b)** $\gamma = 0.4$.

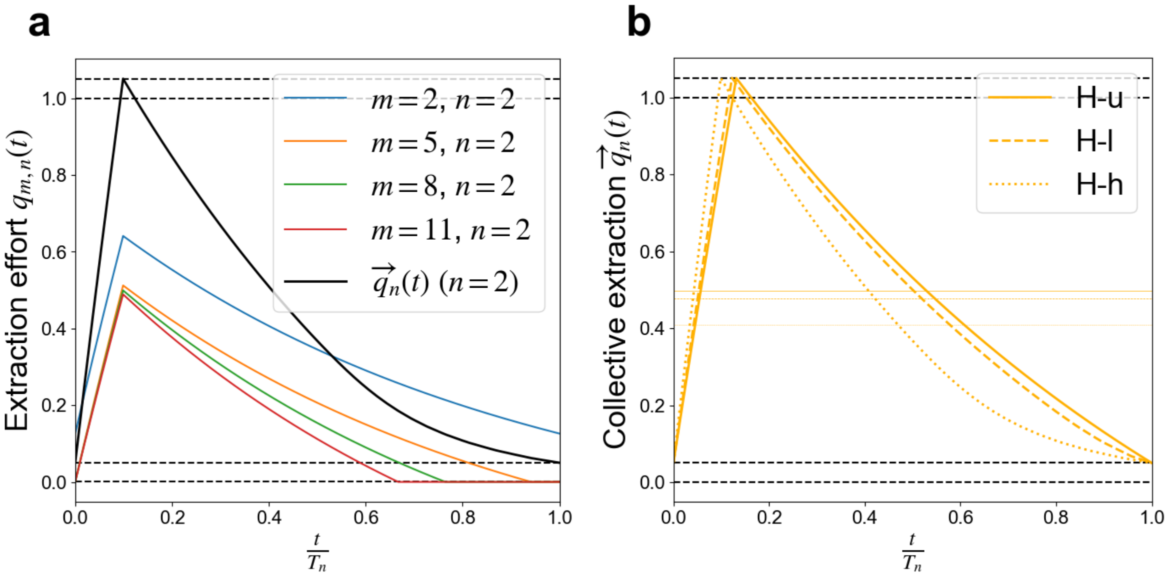


**Figure S24. Increasing depletion and remediation thresholds: $\vec{q}_{\mathrm{R},n} = .05$ and $\vec{q}_{\mathrm{D},n} = 1.05$.** HMF model predicted time evolution of **(a)** Extraction levels for agents of different degrees from a degree-2 source within **H-h** networks, and **(b)** Collective extraction for networks with different types of agent degree heterogeneity and high source degree hetereogeneity (**H**).

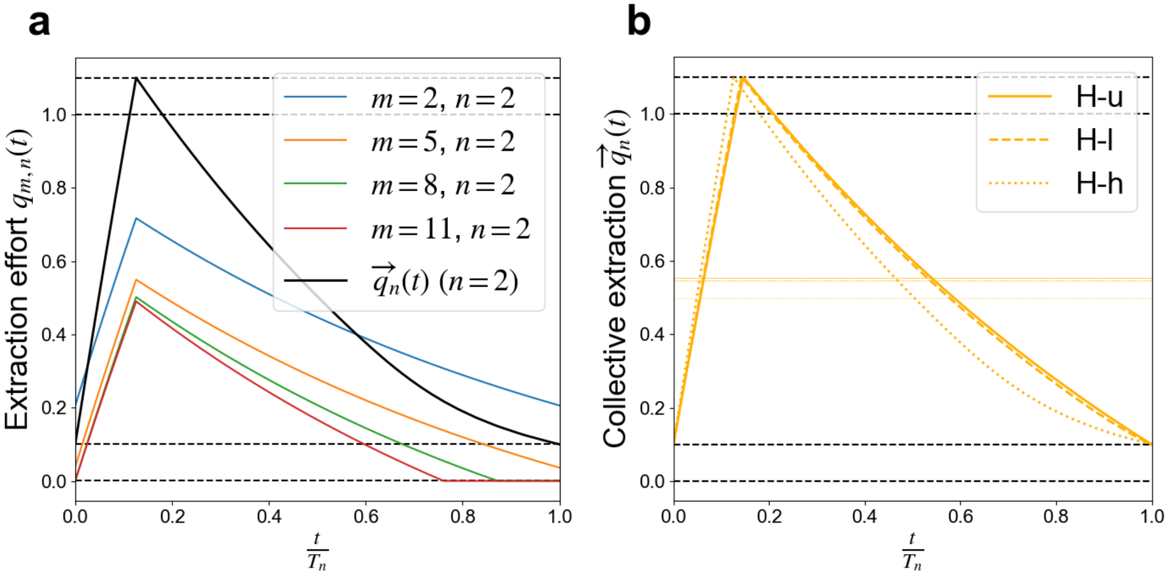


**Figure S25. Increasing depletion and remediation thresholds: $\vec{q}_{\mathrm{R},n} = .1$ and $\vec{q}_{\mathrm{D},n} = 1.1$.** HMF model predicted time evolution of **(a)** Extraction levels for agents of different degrees from a degree-2 source within **H-h** networks, and **(b)** Collective extraction for networks with different types of agent degree heterogeneity and high source degree hetereogeneity (**H**).

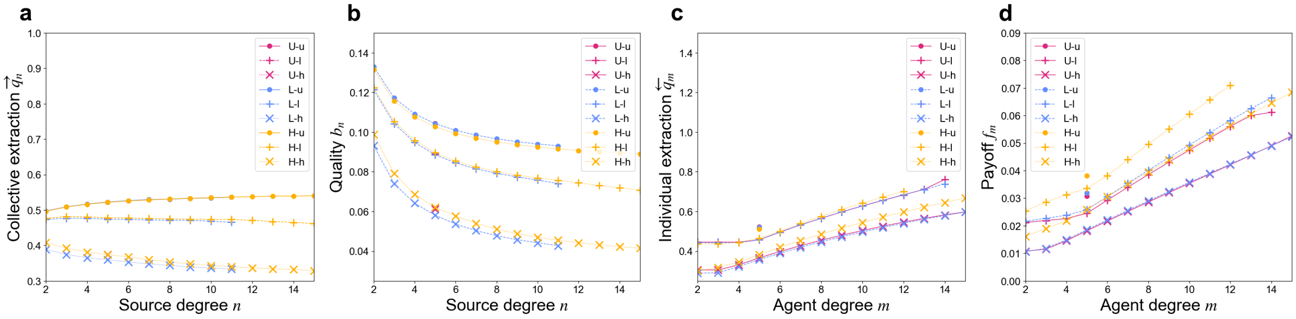


**Figure S26.** **Increasing depletion and remediation thresholds:** $\overrightarrow{q}_{\mathrm{R},n} = .05$ **and** $\overrightarrow{q}_{\mathrm{D},n} = 1.05$**.** Time-averaged quantities from *free adaptation* dynamics estimated using HMF method 2: **(a)** Collective extraction $\overrightarrow{q}_n$, **(b)** Source quality $b_n$, **(c)** Individual extraction $\overleftarrow{q}_m$, and **(d)** Payoffs $f_m$. Other parameter settings remain as before: $\alpha = \beta = 1$ and $\gamma = 0.2$.

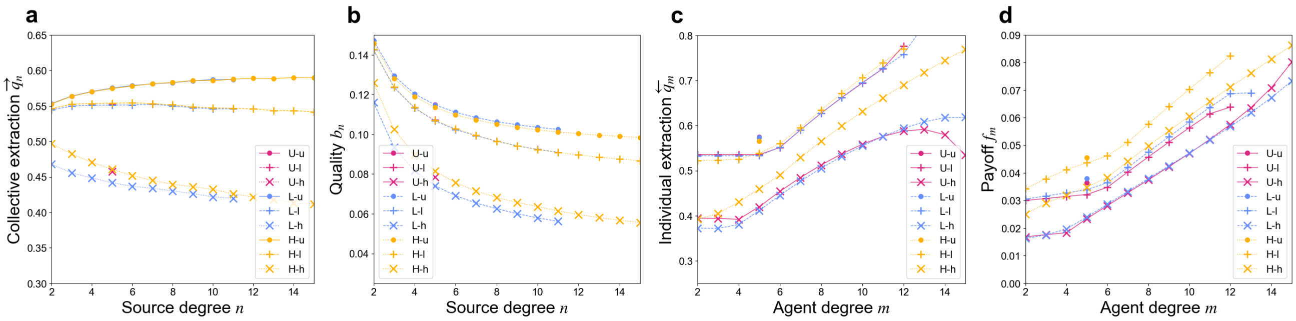


**Figure S27.** **Increasing depletion and remediation thresholds:** $\overrightarrow{q}_{\mathrm{R},n} = .1$ **and** $\overrightarrow{q}_{\mathrm{D},n} = 1.1$**.** Time-averaged quantities from *free adaptation* dynamics estimated using HMF method 2: **(a)** Collective extraction $\overrightarrow{q}_n$, **(b)** Source quality $b_n$, **(c)** Individual extraction $\overleftarrow{q}_m$, and **(d)** Payoffs $f_m$. Other parameter settings remain as before: $\alpha = \beta = 1$ and $\gamma = 0.2$.

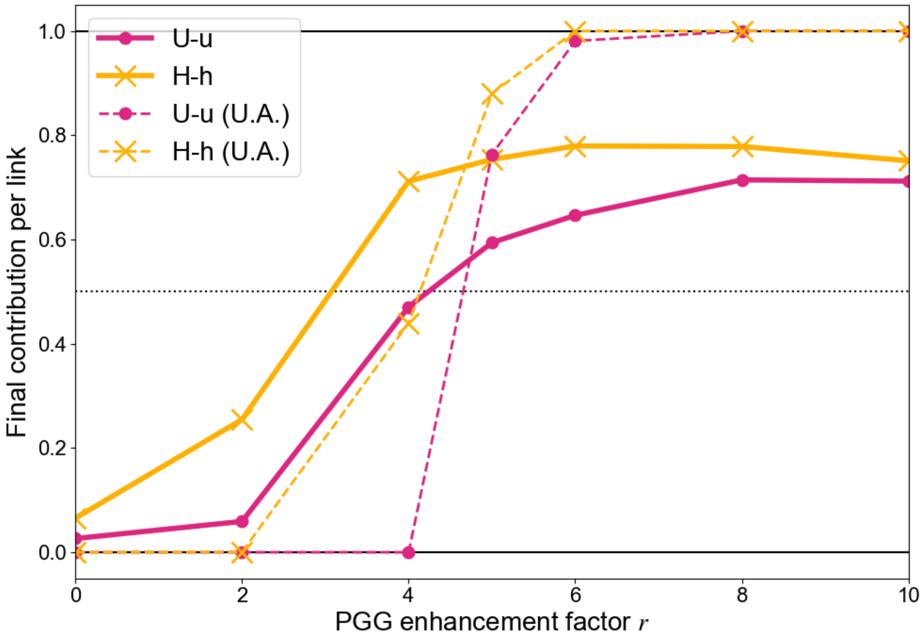


**Figure S28.** Ensemble mean steady-state contribution per link vs. the PGG enhancement factor $r$ for games played with and without a *uniform-allocation* (U.A.) assumption.

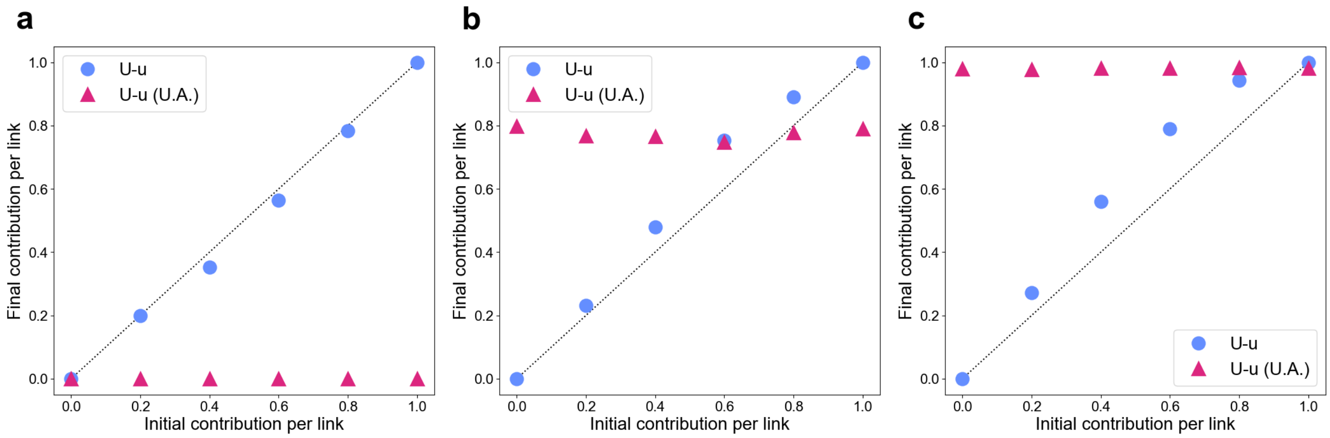


**Figure S29.** Ensemble mean final contribution per link vs. initial contribution per link for sources in games played with and without a *uniform-allocation* (U.A.) assumption on **U-u** networks: (**a**) $r = 4$, (**b**) $r = 5$, and (**c**) $r = 6$.
